# Genome-wide transcription start site mapping of *Bradyrhizobium japonicum* grown free-living or in symbiosis – a rich resource to identify new transcripts, proteins and to study gene regulation

**DOI:** 10.1186/s12864-016-2602-9

**Published:** 2016-04-23

**Authors:** Jelena Čuklina, Julia Hahn, Maxim Imakaev, Ulrich Omasits, Konrad U. Förstner, Nikolay Ljubimov, Melanie Goebel, Gabriella Pessi, Hans-Martin Fischer, Christian H. Ahrens, Mikhail S. Gelfand, Elena Evguenieva-Hackenberg

**Affiliations:** AA Kharkevich Institute for Information Transmission Problems, Russian Academy of Sciences, Bolshoi Karetny pereulok 19, Moscow, 127051 Russia; Moscow Institute of Physics and Technology, Institutskiy pereulok 9, Dolgoprudnyy, Moscow region 141700 Russia; Institute of Microbiology and Molecular Biology, University of Giessen, Heinrich-Buff-Ring 26-32, D-35392 Giessen, Germany; Department of Physics, Massachusetts Institute of Technology, 77 Massachusetts Ave, Cambridge, MA 02139 USA; Agroscope, Institute for Plant Production Sciences, Research Group Molecular Diagnostics, Genomics and Bioinformatics & Swiss Institute of Bioinformatics (SIB), Schloss 1, CH-8820 Wädenswil, Switzerland; Core Unit Systems Medicine, University of Würzburg, Josef-Schneider-Str. 2 Bau D15, D-97080 Würzburg, Germany; Lomonosov Moscow State University, Faculty of Computational Mathematics and Cybernetics, Leninskie Gory, 2-nd educational building, Moscow, 119991 Russia; ETH, Institute of Microbiology, Vladimir-Prelog-Weg 4, CH-8093 Zürich, Switzerland; Department of Bioengineering and Bioinformatics, Lomonosov Moscow State University, Vorobievy Gory 73-1, Moscow, 119991 Russia; Present Address: Institute of Molecular Systems Biology, ETH Zürich, Auguste-Piccard Hof 1, CH-8093 Zürich, Switzerland; Present Address: Department of Plant and Microbial Biology University of Zürich, Zollikerstrasse 107, CH-8008 Zürich, Switzerland

**Keywords:** *Bradyrhizobium*, Nodule, RNA-seq, Transcription start site, Promoter prediction, Proteogenomics, Genome re-annotation, Antisense RNA, Internal transcription start site

## Abstract

**Background:**

Differential RNA-sequencing (dRNA-seq) is indispensable for determination of primary transcriptomes. However, using dRNA-seq data to map transcriptional start sites (TSSs) and promoters genome-wide is a bioinformatics challenge. We performed dRNA-seq of *Bradyrhizobium japonicum* USDA 110, the nitrogen-fixing symbiont of soybean, and developed algorithms to map TSSs and promoters.

**Results:**

A specialized machine learning procedure for TSS recognition allowed us to map 15,923 TSSs: 14,360 in free-living bacteria, 4329 in symbiosis with soybean and 2766 in both conditions. Further, we provide proteomic evidence for 4090 proteins, among them 107 proteins corresponding to new genes and 178 proteins with N-termini different from the existing annotation (72 and 109 of them with TSS support, respectively). Guided by proteomics evidence, previously identified TSSs and TSSs experimentally validated here, we assign a score threshold to flag 14 % of the mapped TSSs as a class of lower confidence. However, this class of lower confidence contains valid TSSs of low-abundant transcripts. Moreover, we developed a *de novo* algorithm to identify promoter motifs upstream of mapped TSSs, which is publicly available, and found motifs mainly used in symbiosis (similar to RpoN-dependent promoters) or under both conditions (similar to RpoD-dependent promoters). Mapped TSSs and putative promoters, proteomic evidence and updated gene annotation were combined into an annotation file.

**Conclusions:**

The genome-wide TSS and promoter maps along with the extended genome annotation of *B. japonicum* represent a valuable resource for future systems biology studies and for detailed analyses of individual non-coding transcripts and ORFs. Our data will also provide new insights into bacterial gene regulation during the agriculturally important symbiosis between rhizobia and legumes.

**Electronic supplementary material:**

The online version of this article (doi:10.1186/s12864-016-2602-9) contains supplementary material, which is available to authorized users.

## Background

In the past five years differential RNA-sequencing (dRNA-seq) has become an essential technology for global analysis of gene expression allowing for the genome-wide mapping of transcriptional start sites (TSSs) [1]. However, such analyses of rhizobia in symbiosis with leguminous plants are still missing. Rhizobia are soil bacteria able to infect plant roots where they induce the formation of root nodules and differentiate into intracellular nitrogen-fixing bacteroids. Bacteroids convert molecular nitrogen into ammonium for the benefit of the plant in exchange for photosynthetically fixed carbon. This microbe-plant interaction is economically important, since rhizobia introduce fixed nitrogen into the global nitrogen cycle and act as an ecologically safe fertilizer. A prominent, rhizobial model organism is *Bradyrhizobium japonicum* USDA 110, a symbiont of the soybean plant *Glycine max* [2, 3].

*B. japonicum* USDA 110 has a large chromosome of 9.1 Mb, with most symbiotic genes clustered in the so-called symbiotic island of 680 kb [4]. Gene-specific deletion and genome-wide transposon mutagenesis studies uncovered many genes relevant for symbiosis and identified their transcriptional regulators [5–11], while microarray-based transcriptome analyses and proteome analyses provided more comprehensive catalogs of differentially expressed genes or proteins that may play a role in the adaptation from free-living conditions to the symbiotic life style within root nodules [12–15]. Previous analysis of tiling microarray data obtained from cells grown in free-living and symbiotic conditions revealed strong transcription in non-annotated regions [13] and validated computationally predicted small non-coding RNAs (sRNAs) [16]. These findings suggest that the initial genome annotation may not be complete, and that additional genes likely remain to be identified.

Recent advances in RNA-seq revealed high complexity of the transcriptional landscape in bacteria, dramatically changed approaches to study regulation of gene expression, and allowed for detection of virtually all non-annotated genes and loci expressed under the conditions of interest [1, 17–21]. Newly detected genes typically comprise short protein-coding ORFs missed in the initial genome annotation, shorter transcripts originating from internal TSS (iTSS), and *cis-* or *trans*-encoded sRNAs that are hard to detect by other methods [1, 19, 20]. Previously, several *trans-*encoded sRNAs have been detected in *B. japonicum* USDA 110 [16], but genome-wide detection of sRNAs has not been performed so far in this organism.

The differential RNA-seq (dRNA-seq) method [19] relies on the enrichment of 5′-ends of primary, non-processed transcripts. This allows for accurate genome-wide determination of transcription start sites (TSSs), and thus the primary transcriptome under specific environmental conditions. The first global TSS mapping was performed manually for the relatively small genome of *Helicobacter pylori* [19]. Subsequently, TSSs in larger bacterial genomes were mapped, in several cases aided by the use of computational methods [20, 22–25], but global mapping of TSSs is still challenging.

The detection of an authentic TSS (a 5′-end of a primary transcript) implies the presence of a promoter in its upstream region. Bacterial RNA polymerase recognizes promoters with the help of sigma factors, which, based on homology and mechanism of action form two families, σ^70^ and σ^54^ [26]. *B. japonicum* USDA 110 harbors 21 members of the σ^70^ family and two σ^54^ paralogs [4, 27, 28]. In exponentially growing, free-living cells, the expression of housekeeping *B. japonicum* genes relies on the σ^70^-type σ-factor RpoD, which binds to conserved boxes located 10 and 35 bp upstream of the TSS [29]. Functions and promoter motifs were investigated for only a few of other σ^70^ family members [28, 30–32]. In symbiosis, many genes for nitrogen fixation and associated functions are under the control of σ^54^ (RpoN), which enables RNA polymerase to recognize promoters with conserved GG and GC boxes located at positions –24 and –12 relative to the TSS, respectively [27, 33]. Known promoter motifs in *B. japonicum* were identified by analyzing DNA sequences upstream of a limited number of experimentally detected TSSs. A global TSS map containing nearly all TSSs active under certain growth conditions and thus allowing genome-wide analysis of potential promoter regions [24, 34] will greatly facilitate future studies on gene regulation in *B. japonicum* and related bacteria.

The aim of this study was to generate dRNA-seq data of *B. japonicum* USDA 110 grown free-living or in symbiosis with soybean to be used for genome-wide mapping of TSSs and promoters, and for identification of new genes. To perform global mapping of TSSs, we developed a TSS-identification tool that uses machine-learning approaches to propagate expert knowledge initially applied to a subset of the data. For identification of new protein-coding genes, we used a proteogenomics approach. Furthermore, we used our condition-specific TSS map to predict and map promoters by a new algorithm, which is publicly available. Finally, we provide an updated and extended genome annotation with mapped promoters, TSSs and terminators in the generic feature format 3 (gff) and the Gene Bank sequence format (gbk). We expect that these data will serve as a useful resource both for detailed analysis of specific genes and for systems biology studies of the symbiosis between rhizobia and legumes, as well as for future annotations of bacterial genomes.

## Results and discussion

### dRNA-seq and read mapping

To establish a comprehensive, condition-specific TSS map of *B. japonicum* USDA 110, we performed dRNA-seq with total RNA isolated from bacteria exponentially growing in oxic liquid cultures (hereafter referred to as Free) and from soybean root nodules (hereafter referred to as Nod). Following the dRNA-seq protocol, one half of the RNA samples was treated with terminal exoribonuclease (TEX), which degrades 5′-monophosphorylated transcripts. This resulted in the enrichment of primary, 5′-triphosphorylated transcripts (+ library). The other half remained untreated (– library). Enrichment of a 5′-end in the (+) library indicates a TSS [19].

**Fig. 1:**
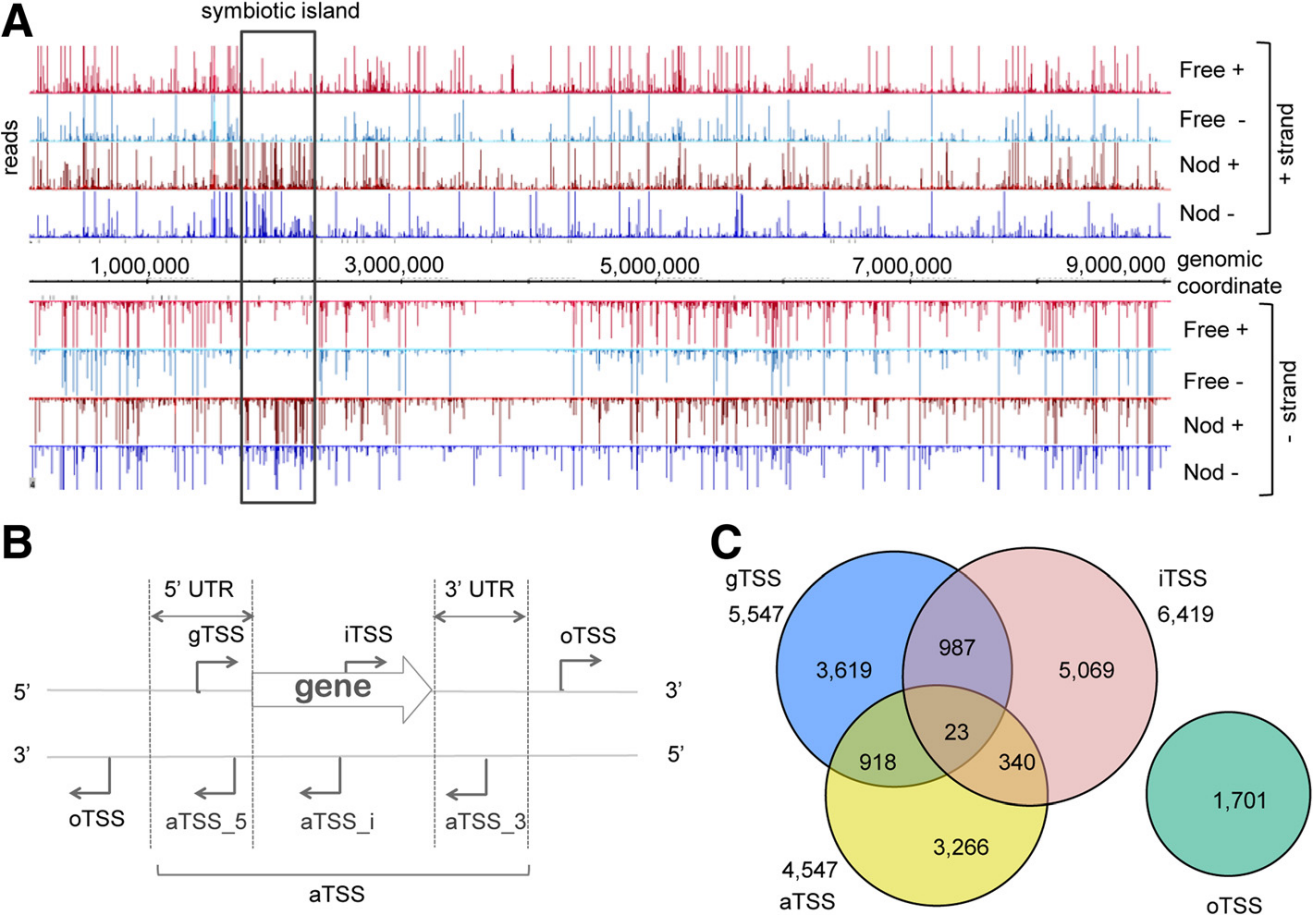
Differential RNA-seq of *B. japonicum* USDA 110 and TSSs mapping. **a** cDNA reads mapped to the 9.1 Mb genome. RNA was isolated from exponentially growing, free-living cells (Free) in liquid cultures and from nodules (Nod). RNA samples were treated (+) or not treated (–) with TEX. As expected, there are more reads in the Nod libraries mapped to the symbiotic island compared to the Free libraries (*framed*). All libraries were adjusted to the same scale (0 to 300 reads). **b** Categories for TSS annotation based on the genomic context: gTSS, TSS of an annotated gene; iTSS, internal TSS (located in an annotated gene) in sense orientation; aTSS, antisense TSS (aTSSs are divided in the following sub-categories: aTSS_5, aTSS located in a region defined as 5′ UTR; aTSS_i, internal aTSS located inside an annotated gene; aTSS_3, aTSS located in a region defined as 3′ UTR); oTSS, orphan TSS, all TSSs which do not fall in the categories mentioned above. **c** Venn diagram, showing distribution of the TSSs among the categories gTSS, iTSS, aTSS and oTSS

Overall 8,883,409 reads were obtained, 4,109,857 (46 %) of which were successfully mapped to the *B. japonicum* genome (Fig. 1a; Additional file 1: Table S1). For the Free sample, 94 % of the reads were mapped to the *B. japonicum* genome, while for the Nod sample, only 18 % were mapped due to a large fraction of soybean transcripts (Additional file 1: Table S1). In a previous dRNA-seq study with the plant pathogen *Xanthomonas campestris*, the majority of reads from both (+) and (–) libraries mapped to rRNA loci (68 and 63 % respectively; [22]). In our study, however, ribosomal RNA was considerably depleted after the TEX treatments: in the (+) libraries, 15 % (Nod) and 19 % (Free) of the reads belonged to rRNA genes, compared to 69 % (Nod) and 59 % (Free) of the reads in the (–) libraries. Since bacterial rRNAs have processed 5′-ends [35], this indicates that in our experiments the TEX treatment effectively depleted processed transcripts.

### TSS detection by machine learning

To map TSSs, we used a Support Vector Machine (SVM) method of machine learning. We defined candidate TSS as a peak of the salience function (Methods; Additional file 2: Figure S1) with matching coordinates in the (+) and (–) libraries under at least one of the experimental conditions (Free or Nod), and applied SVM independently to Free and Nod pairs of the (+) and (–) libraries. The SVM algorithm learns a model of a TSS from a small, expert-curated training set, and applies it to the whole genome, allowing for automatic TSS detection in large genomes (for details on the algorithm, see Methods). Two training sets, comprising 202 peaks in Free and 182 peaks in Nod, were used to derive a SVM model, which was then applied to 30,162 and 6454 peaks in Free and Nod, respectively. After SVM filtering, we obtained 17,205 putative TSSs in Free and 4558 in Nod; 2837 scored positively in both conditions (Additional file 2: Figure S2).

Additional filtering was applied to putative TSSs located within coding regions of expressed genes (see Methods). In total, 15,923 TSSs active in Free and/or Nod were retained after these filtering procedures and mapped to the *B. japonicum* genome (see Additional file 3: Table S3). This number is comparable to 17,001 TSSs mapped in the smaller genome (6.7 Mb) of the alfalfa symbiont *Sinorhizobium meliloti*, detected under several growth or stress conditions in liquid cultures [24].

To assess the reliability of the SVM-based TSS mapping, we compared our data to previously published results. Our data matched 35 out of 38 previously determined TSSs of genes expressed under symbiotic conditions or in free-living cells, i.e., under the conditions investigated in this study (Additional file 3: Table S4). Well-known examples are genes blr1769 (*nifH* encoding the dinitrogenase reductase) and blr1759 (*nifB* encoding a nitrogenase cofactor biosynthesis protein). As expected, transcripts of these genes were detected only in bacteroids and the respective TSSs Bja_TSS_3777 and Bja_TSS_3758 were mapped at previously determined genomic positions 1,928,416 and 1,921,754 [36, 37]. Known TSSs induced under conditions not relevant to our study either did not pass our stringent filtering criteria (e.g., TSS T_2_ of the heat shock sigma factor gene *rpoH*_2_ at genomic position 8,074,642 used predominantly at high temperature, [30]), or were scored but had low peaks consistent with low expression of the corresponding genes (*ecfQ*, *ecfF*, bsl1652; [13, 28, 38]). These and additional examples summarized in Additional file 3: Table S4 demonstrate the quality of TSS mapping based on dRNA-seq and machine learning.

### Genome re-annotation and UTR length determination

To assess the coding and non-coding transcript repertoire, it is convenient to group TSSs in categories based on their genomic context. Since the TSS categorization critically depends on the genome annotation and 5′- and 3′-UTR definition, we first addressed these issues in more detail. We performed genome re-annotation using Integrated Services of Genomics Analysis (ISGA) [39] and found that it better conforms to the dRNA-Seq data than the present RefSeq annotation [4] (see Methods and Additional file 4). The ISGA annotation, in which the original gene identifiers (locus tags) of the RefSeq annotation were preserved, was the basis of annotation files in the gbk and gff formats (Additional files 5 and 6), in which additional features were included (see below; see also Additional file 4).

Using this annotation, we explored the genome-wide distribution of distances between mapped TSSs and annotated genes in order to set 5′- and 3′-UTR maximal lengths for TSSs classification purpose. First, we analyzed the distribution of predicted 5′-UTR (leader) lengths, i.e., the distance from a TSS to the start of the downstream gene or ORF. We found that 5′-UTRs are typically 20–40 nt long and rarely exceed 200 nt, and thus the maximal length of the 5′-UTRs was set to 200 nt (for details see Additional file 4). The estimation of a maximal 3′-UTR length was based on the consideration that as an antisense RNA should overlap with the respective mRNA, aTSSs should be often located at a short distance from the mRNA. Indeed, the distribution of distances between stop codons and downstream antisense TSSs showed a prominent peak at 20–30 nt downstream of stop codons (see Additional file 4). This intriguing finding suggests that RNA-based regulation at the 3′-end of ORFs plays an important role in *B. japonicum.* Antisense RNA overlapping the stop codon may influence translation and stability of the cognate mRNA, as well as transcription termination [40]. For TSSs classification purpose, we set the maximal length of 3′-UTRs to 100 nt.

### TSS categorization

The mapped TSSs were categorized as shown in Fig. 1b. The distribution of 15,923 TSSs among the categories gTSS (TSS of an annotated gene), aTSS (antisense TSS), iTSS (internal TSS in the sense orientation) and oTSS (orphan TSS) categories is shown in Fig. 1c and described below. All TSSs are annotated in Additional files 5 and 6, and their categories, SVM scores and peak heights are listed in Additional file 3: Table S3. Note that as 5′-UTRs and 3′-UTRs of adjacent genes may overlap, many TSSs are assigned to multiple categories simultaneously. Accordingly, 20,071 TSSs are listed in Additional file 3: Table S3.

#### gTSSs

We detected gTSSs upstream of 46 % of all annotated genes. This probably underestimates the fraction of the expressed genome since many bacterial mRNAs are polycistronic. Assuming that all genes in an operon are expressed, if they are located downstream of a mapped TSS, we estimated that at most 62 % of the annotated *B. japonicum* USDA 110 genes were expressed under our experimental conditions (Free and/or Nod). Typically, gTSSs are located 20–40 nt upstream of the start codon of an annotated gene (Additional file 4). Additionally, gTSSs were annotated for 7 of 12 operons predicted to be preceded by riboswitches [41]; in this case the leader regions were allowed to be longer than 200 nt. Further, 320 gTSSs mapped 0 to 10 nt upstream of annotated start codons, with 192 mapped exactly at the start codon, suggesting that they correspond to leaderless mRNAs.

#### iTSSs

iTSSs are the most abundant category of TSSs in this study (40 % of all TSSs are potential iTSS; Fig. 1c). A similarly high fraction of iTSSs was detected in *S. meliloti* (45 %, [24]), while in *Synechocystis* sp*.*, *X. campestris* and *H. pylori* the percentage of iTSSs was 29, 22 and 19 %, respectively [19, 20, 22]. A TSS mapping inside an annotated ORF may originate from (i) a gTSS of a gene with a misannotated start codon [42]; (ii) a gTSS of an overlapping gene transcribed in the same direction or of a sub-operon; or (iii) a monophosphorylated 5′-end of an RNA degradation product that was not efficiently digested by the TEX. We analyzed the distribution of iTSSs (see Additional file 4) and found that the vast majority of iTSSs is distributed uniformly in genes, thus representing genuine iTSS candidates. In addition, this analysis also revealed clustering in the first 30 bp (suggesting that some genes are shorter than annotated) and, although less prominent, in the last 30 bp of genes (indicating that some iTSSs are probably TSSs of downstream genes) (see Additional file 4). Recent studies confirmed the presence of iTSSs in other bacteria and suggested a mechanism for internal transcription initiation by elongating RNA polymerase complexes that still contain the σ^70^ factor [43, 44]. However, 53 % of the iTSSs map to genes or operons without assigned gTSSs (see Additional file 1: Table S2). According to dRNA-seq data, these genes are not transcribed, suggesting that the majority of the iTSSs does not correspond to degraded mRNA. Nevertheless, we cannot exclude that some of the mapped iTSSs represent stable degradation products of primary transcripts whose 5′-ends are degraded very quickly thereby preventing the detection of their genuine TSSs.

#### aTSSs

Our data suggest substantial antisense transcription in *B. japonicum* USDA 110: 28 % of all detected TSSs are aTSSs (Fig. 1c) and they map opposite to 29 % of annotated genes (see Additional file 1: Table S2) including symbiotically important genes like *nifB*, *nifD*, *nifH* and *nifW* (Additional file 2: Figures S3 and S4). We note that 52 % of annotated genes with aTSSs do not have gTSSs and are not expressed under the tested experimental conditions (see also Additional file 1: Table S2), suggesting differential expression of asRNAs and their complementary mRNAs. Previous studies in other bacteria suggested that differences in the expression patterns of genes for asRNAs and cognate mRNAs prevent unnecessary mRNA transcription under short-term environmental changes [45–47]. However, despite the widespread occurrence of asRNAs in bacteria, only for a small subset of asRNA physiological roles were established [40].

#### oTSSs

In addition to the above TSSs associated with annotated genes, 1701 oTSSs were detected (Fig. 1c). 11 % of all *B. japonicum* USDA 110 TSSs belong to this category, compared to 1.6 % oTSSs in *H. pylori* [19], 5.3 % in *X. campestris* [22], 3.5 % in *S. meliloti* [24] and 9 to 11 % in cyanobacteria [20, 48]. The category oTSS corresponds to non-annotated transcripts, e.g., *trans*-encoded, regulatory sRNAs [19, 20]. The definition of oTSS depends on the threshold length set for 5′- and 3′-UTRs, which differ in individual studies: here we set 5′- and 3′-UTRs of *B. japonicum* to 200 and 100 nt, respectively, whereas these elements were longer (300 nt each) or shorter (100 nt (5′-UTR) and 50 nt (3′-UTR)) in comparable studies with *S. meliloti* [24] and *Synechocystis* [20], respectively. This suggests that the higher proportion of oTSSs in *B. japonicum* reflects, at least partly, differences in the UTR definitions. Finally, oTSSs and also iTSSs could originate from pervasive transcription, which was recently suggested to play an important role in gene regulation and genome evolution in prokaryotes and eukaryotes [49].

### TSSs in free-living cells and in bacteroids

**Fig. 2:**
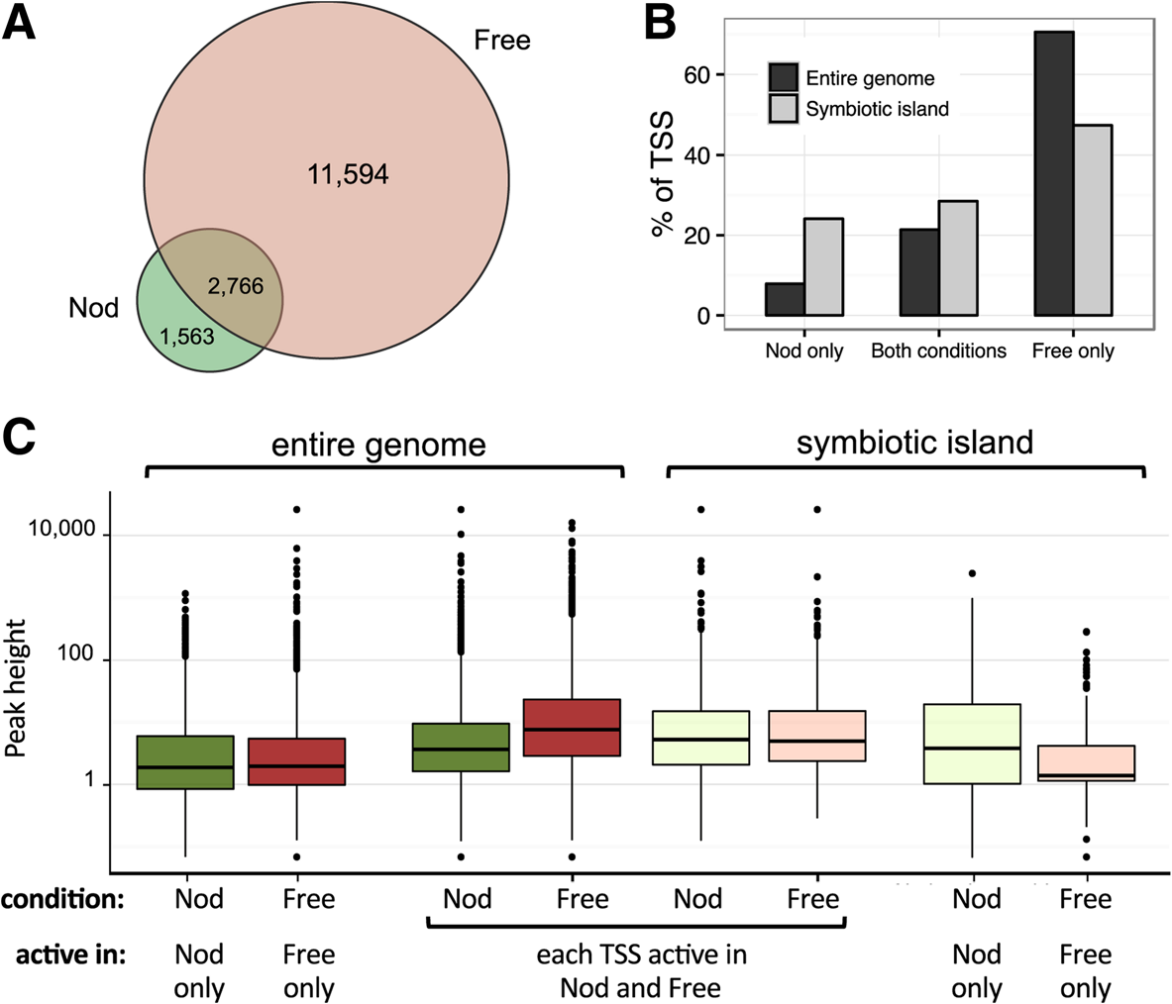
TSSs in free-living cells and in bacteroids. **a** Venn diagram showing the numbers of TSSs detected in Free and in Nod. **b** Diagram showing the percentage of TSSs mapped to the symbiotic island or to the entire genome and belonging to one of the following three categories: detected only in Nod, detected in both conditions and detected only in Free. Light bars, symbiotic island; dark bars, entire genome. **c** Boxplots showing the distribution of peak heights (logarithmic scale) in Nod and Free for TSSs mapped to the entire genome or to the symbiotic island, as indicated. Peaks corresponding to TSSs active in both conditions (see **b**) were assessed separately in the Nod and in Free libraries. Horizontal lines are the median values

We used the dRNA-seq analysis to compare the primary transcriptome of free-living *B. japonicum* to that of bacteroids in soybean root nodules. Of 15,923 TSSs identified in this study, 14,360 were detected in Free and 4329 in Nod, with 2766 being detected under both conditions (Fig. 2a). This is in agreement with previous transcriptomics data [13] showing that a much lower number of genes (2780) were expressed during symbiosis compared to free-living conditions (5439 genes) and can be explained by the non-dividing and thus transcriptionally less active state of nitrogen-fixing bacteroids [50, 51]. The data also indicate one advantage of a dRNA-Seq approach: due to the ability to directly map reads against two reference genomes, more transcripts were identified in symbiosis by dRNA-seq compared to the hybridization-based microarray analysis [13], where these signals cannot be separated in a similar manner.

We mapped 1485 TSSs to the symbiotic island where many genes essential for the symbiotic nitrogen fixation are located. As expected, the symbiotic island is enriched in TSSs active only in Nod: it spans 7 % of the genome, and contains 7 % of annotated genes and 9 % of mapped TSSs, but harbors 24 % of the TSSs, which were detected only in Nod. Despite this enrichment, most TSSs in the symbiotic island were detected only in Free (Fig. 2b). Together with the results from Fig. 1a where genes most strongly transcribed in symbiosis mapped to the symbiotic island, this suggests that TSSs detected only in Free and mapping in this region are preferentially weak. Analysis of the distribution of peak heights both in the symbiotic island and in the entire genome indeed revealed that in the symbiotic island the heights of peaks detected only in Free were much lower than the heights of peaks detected only in Nod (Fig. 2c).

Our dRNA-seq and TSS mapping results are in agreement with previous microarray gene expression data [13]. We mapped gTSSs to 68 % of the 2780 genes previously identified as expressed in symbiosis [13]. Moreover, TSSs of numerous genes shown previously to be up-regulated during symbiosis (e.g., *nif* genes encoding nitrogenase and associated functions, *modB*, blr1853 and blr2143 [13]) belong to the TSSs which were mapped only in Nod (Additional files 5 and 6).

### Protein translation evidence for TSSs data

While dRNA-seq data provide a global picture of transcription, proteomics data contribute direct evidence on transcripts translated into protein products [52]. Though massive efforts are required to describe a complete condition-specific proteome [53–55], this approach would provide a best possible complementary data set to a global condition-specific TSS map.

To explore additional evidence for translation of transcripts with TSSs identified here, we re-analyzed existing proteomics data of *B. japonicum* USDA 110 grown under free-living conditions in rich PSY medium or in minimal medium [56], and in symbiosis with soybean (*G. max*) [15], cowpea (*Vigna unguiculata*) or siratro (*Macroptilium atropurpureum*) [57]. For this, we devised a novel variant of a proteogenomics approach that relies on generating an extended protein search database guided by the TSS evidence for (i) ORFs missed in the original RefSeq annotation, including short ORFs which are typically under-represented in genome annotations [58], here taken from the ISGA annotation (see Additional files 5, 6 and 7), (ii) ORFs that are longer or shorter compared to the RefSeq annotation, and (iii) evidence for proteins encoded by transcripts originating from an iTSS.

**Fig. 3:**
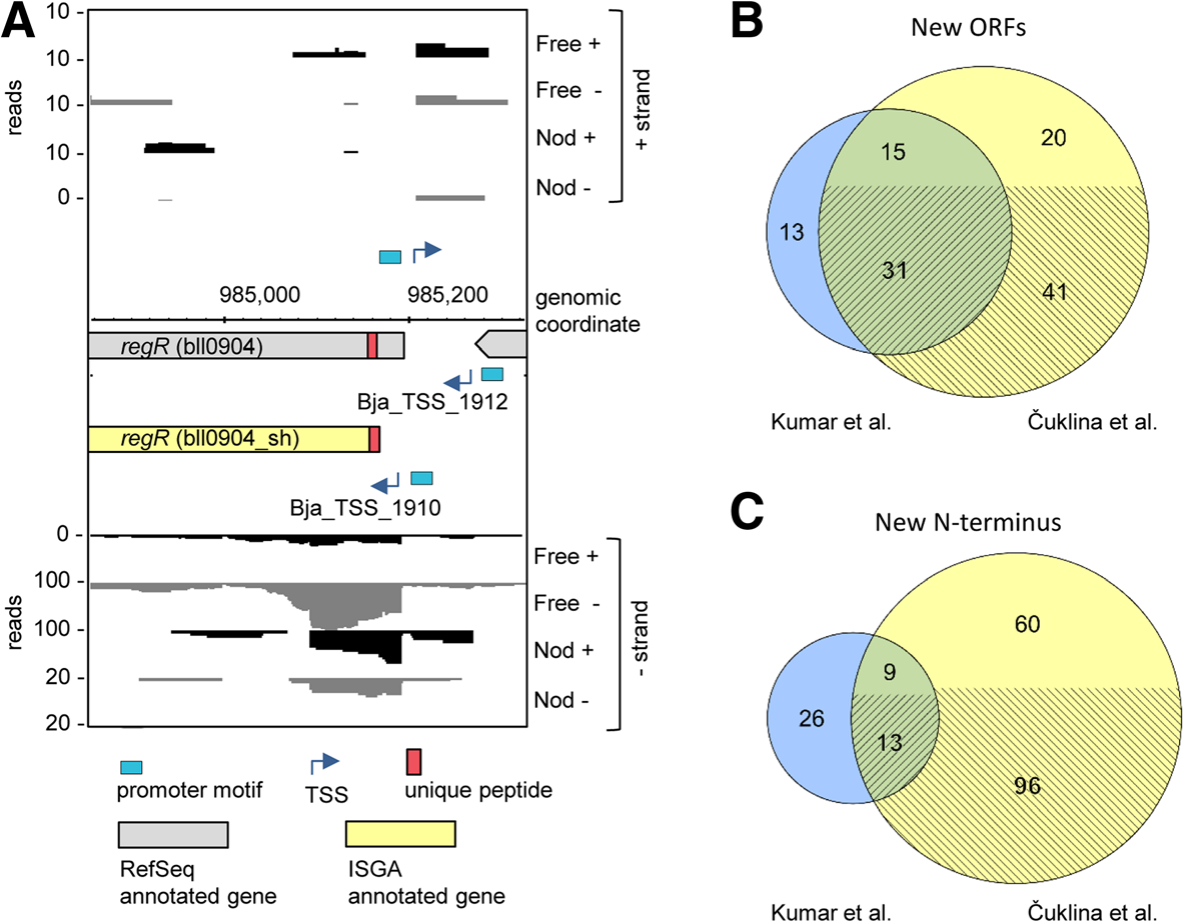
Combined transcriptomics and proteomics evidence for shorter or longer protein forms and new ORFs. **a** Genome region of bll0904 encoding RegR (grey box, annotated by RefSeq). Predicted promoter motifs (*blue box*) and TSS (*blue arrows*) along with dRNA-seq evidence from Free and Nod indicated the existence of a shorter *regR* form (yellow box, ISGA annotation), whose expression is further confirmed by proteomics evidence. Unique peptides (*red boxes*) that provide evidence for both the long and the new, shorter protein form were observed; for clarity, the remaining peptide evidence is not shown. The TSS Bja_TSS_1912 preceding the long form of the *regR* gene was mapped at genomic position 985,265. The shorter form of the gene is preceded by Bja_TSS_1910 mapped at genomic position 985,187 (Additional file 3: Table S3; Additional file 7: Table S5). Note the different scales of individual libraries. For further details, see legend of Fig. 1a. **b** Venn diagram with the number of novel ORFs identified by a previous proteogenomics study by Kumar et al. [61], and our proteogenomics approach that is additionally guided by TSS evidence (see Methods). **c** Venn diagram with the number of new N-termini identified by Kumar et al. [61], who had searched for evidence for longer proteins, and our study, that also identified shorter protein forms. For **b** and **c**, the number of ORFs supported by a TSS is represented by the cross-hatched lower parts of the circles

In conditions corresponding to our dRNA-seq analysis, we were able to provide evidence for 3553 protein groups, namely 3176 in rich medium (corresponding to Free in dRNA-seq) and 2063 in symbiosis with soybean (corresponding to Nod in dRNA-seq; Table 1). This included evidence for 78 new ORFs from the ISGA annotation (Table 1, columns 3, 4 and 5). Furthermore, we provide evidence for 109 shorter and 32 longer forms compared to the RefSeq annotation, and 12 proteins corresponding to transcripts with iTSSs. Notably, for 7 proteins we detected peptides confirming that both longer and shorter forms are translated. As one example, we show the combined dRNA-seq and proteomics evidence for two protein isoforms of RegR, a response regulator important for nitrogen fixation (Fig. 3a; [9]). Both isoforms were detected in free-living and symbiotic conditions (Additional file 7: Table S5). The ISGA genome annotation, promoter and TSS mapping, combined with proteomics data support expression of an alternative, shorter form of RegR.

**Table 1:** Proteomics and dRNA-seq evidence for new ORFs and longer or shorter proteins than originally annotated

Protein class	Proteins in search database	Free and Nod (dRNA-seq support)	Free (Free dRNA-seq support)	Nod (Nod dRNA-seq support)	Over all conditions
Annotated in RefSeq and ISGA	4749	3187 (1958)	2875 (1747)	1893 (711)	3608
New in ISGA	1391	78 (53)	64 (46)	46 (22)	107
Shorter in ISGA	2857	109 (71)	92 (60)	41 (22)	139
Longer in RefSeq	2857	108	86	51	144
Longer in ISGA	194	32 (19)	31 (16)	11 (3)	39
Shorter in RefSeq	194	-	-	-	0
iTSS ORFs	5894	12 (12)	10 (10)	4 (0)	14
RefSeq only	517	27	18	17	39
Total	18,653	3553 (2113)	3176 (1879)	2063 (758)	4090

Numbers of proteins originally annotated in RefSeq and/or in our ISGA re-annotation are listed in column 2. Numbers of proteins identified in rich PSY medium or in symbiosis with soybean, i.e., the Free and Nod conditions studied here with dRNA-seq, are listed in columns 3-5, along with dRNA-seq support (without considering operons); column 6 “Over all conditions” refers to protein identifications in all 5 conditions - growth in rich and minimal medium, and symbiosis with soybean, cowpea or siratro. The respective protein IDs are also available in Additional file 7: Table S5

Overall, when including data from symbiosis with other host plants and growth in minimal medium, we identified 4090 proteins (Table 1, column 6). Among them were (i) 107 new proteins (72 with TSS support), identified over all conditions, which correspond to new ISGA genes, and (ii) 39 of the 517 proteins encoded by genes exclusively found in the RefSeq annotation (Additional file 7: Table S5). This shows that no single annotation contained all proteins identified here, and supports the need to integrate protein expression evidence into the genome annotation process [52, 59, 60]. The genes of these 39 proteins were included in our re-annotation files (Additional files 5 and 6).

Finally, we compared the newly identified proteins with those found in a previous proteogenomics study on *B. japonicum* USDA 110 [61]. Based on integration of our global TSS map data and the extensive *B. japonicum* protein dataset comprising 4090 proteins detected over all conditions, we found 61 proteins (41 with TSS support) not identified previously [61] (Fig. 3b). In addition, we provide evidence for 178 shorter or longer proteins (109 with TSS support), compared to 48 longer proteins identified in the previous study [61] (Fig. 3c and Additional file 7: Table S6). These results show that integrating dRNA-Seq data with a proteogenomics approach can provide additional value for genome annotation.

### Prediction of promoter motifs using the TSS map

**Fig. 4:**
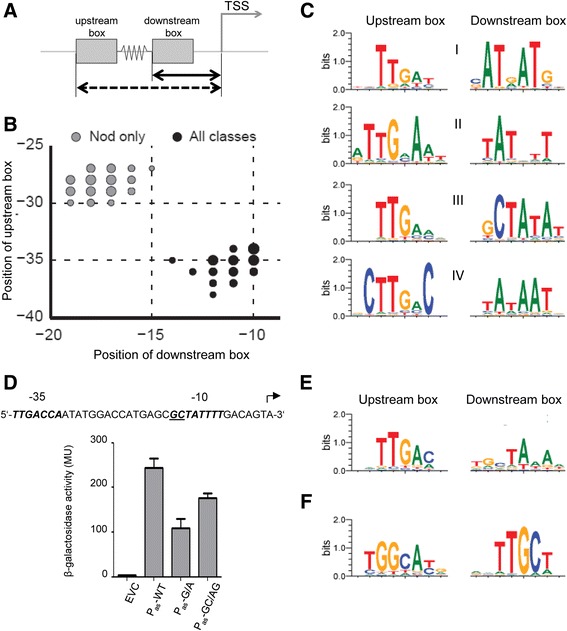
Promoter motifs predicted *de novo* based on genomic regions upstream of mapped TSSs. **a** Schematic representation of a bacterial promoter: upstream 6-mer box (*distance to TSS denoted with dashed arrow*), downstream 6-mer box (*distance to TSS denoted with solid arrow*). **b** Promoter location distribution. Location is a combination of positions of an upstream box (length of dashed arrow in panel **a** and a downstream box (length of solid arrow in panel **a**. Position is shown relative to TSS, in 5′ to 3′ direction. Locations are shown for TSS upstream regions of all TSSs (“all classes”, *black circles*), and TSSs active in nodules only (“Nod only”, *grey circles*). Circle size is proportional to number of motifs mapping in specific positions (location). **c** Four motifs (I to IV) similar to typical RpoD-dependent promoters were found when the regions upstream of all TSSs were analyzed. **d** A predicted RpoD-dependent promoter (P_as_) with an extended downstream (–10) box (motif III in panel **a** was verified experimentally. The sequence upstream of the mapped TSS Bja_TSS_3939 (*marked with a bent arrow*) is shown. The –35 and –10 box of the predicted P_as_ are in bold and italics, and the GC extension of the –10 box is underlined. The 63 nt region upstream of the TSS was transcriptionally fused to the *lac* operon and beta-galactosidase activity measurements in *B. japonicum* were performed. The introduced mutations in the GC extension are indicated. Shown are results from three independent experiments with technical duplicates with error bars depicting the standard deviation. **e** A motif detected upstream of leaderless mRNAs. **f** A motif similar to RpoN-dependent promoters was found, when the regions upstream of TSSs detected in Nod only were analyzed

A genome-wide TSS map can also be exploited to predict specific promoter motifs. Most known bacterial promoters are composed of two conserved sequence motifs (upstream and downstream boxes, Fig. 4a) separated by a spacer of conserved length, which are located at a defined distance upstream of the TSS and recognized by the RNA polymerase sigma subunit [26]. To identify such promoter motifs *de novo*, we developed a computational algorithm, which analyzes the upstream regions of the detected TSSs. To discover promoter motifs, we described promoter sequences using “patterns”, pairs of 6-mers at certain distance upstream of a TSS, separated by a spacer of conserved length (see Methods and Additional file 4). We identified 6-mers co-occurring more frequently than expected by scanning all possible 6-mers at all positions (see Additional file 4). Then we separated distinct groups of overrepresented motifs by the principal component analysis (PCA). We performed this analysis for all TSSs, for TSSs detected only in Nod, in both conditions and only in Free, and for TSSs of the 320 leaderless transcripts (Fig. 4 and Additional file 2: Figure S5).

Motifs overrepresented in the total pool of TSS upstream regions map to positions –35 and –10 upstream of TSSs (Fig. 4b) and are shown in Fig. 4c (motifs I to IV). When upstream regions of TSSs detected either in both conditions or only in Free were analyzed, essentially the same motifs were found (Additional file 2: Figure S5). These motifs are similar to the typical –35 and –10 box-containing promoters recognized by the *E. coli* housekeeping σ factor RpoD [26]. In addition to the TTG-N(16-18)-TATA consensus, each of the motifs has additional specific sequence features (Fig. 4c).

The extended -10 box GCTATA of motif III was previously found in promoters of genes involved in biosynthetic or housekeeping functions in the α-proteobacterium *Caulobacter crescentus* [62]. We confirmed experimentally the functional importance of this GC extension in the predicted promoter P_as_ of asRNA AsR1-blr1853 in *B. japonicum* (Fig. 4d; see also Fig. 9c below). The comparison of the empty vector control strain (EVC) to the strain containing the wild-type sequence (P_as_-WT) confirmed promoter activity in the cloned region. The activity was reduced by G → A and GC → AG mutations in the GC extension of the –10 box.

The CTTG in the –35 box (Fig. 4c, motif IV) and the C upstream of the –10 box (motif III) were found in RpoH-dependent promoters in *S. meliloti* [63], suggesting that motif IV and/or III could be recognized by RpoH which is another member of the σ^70^ family of sigma factors. In *B. japonicum*, *rpoH*_*2*_ is one of three *rpoH* genes, and essential for growth under standard laboratory conditions [30]. It is possible that RpoH_2_-dependent genes, which are expressed under the conditions applied for our dRNA-seq analysis, are associated with promoter motifs III or IV (Fig. 4c).

The motif found by the analysis of regions upstream of leaderless mRNAs has an extended –10 box consensus TGnTA (Fig. 4e). A similar motif is present in promoters of leaderless bacteriophages genes [64]. When we mapped this motif back to the genome, we found it upstream of 2560 TSSs, probably due to its similarity to RpoD-type motifs (Fig. 4c and e). Consistently, both types of putative promoters, RpoD-like and leaderless-like, were mapped upstream of 2106 TSSs in our experiment. Though leaderless bacterial mRNAs have been shown to be involved in stress defense [65], no specific promoters are described in the literature for this type of genes.

The motif found by the analysis of regions upstream of TSSs detected only in Nod is highly similar to RpoN-dependent promoters (Fig. 4b and f), which are characteristic for many nitrogen fixation genes [33, 66]. Thus our results are consistent with the important role of RpoN (σ^54^) for nitrogen fixation and life inside the nodule [27].

Overall, we predicted 4007 RpoD-like and 1201 RpoN-like promoters upstream of TSSs (predicted promoters are listed in Additional file 8: Tables S7, S8 and S9; for details on the mapping of promoters upstream of TSSs, see Additional file 4). For 305 TSSs, the simultaneous presence of σ^70^- and σ ^54^-type promoters was detected (Additional file 9). Candidate promoter sequences and their genomic coordinates are included in the annotation files gff and gbk (Additional files 5 and 6).

**Fig. 5:**
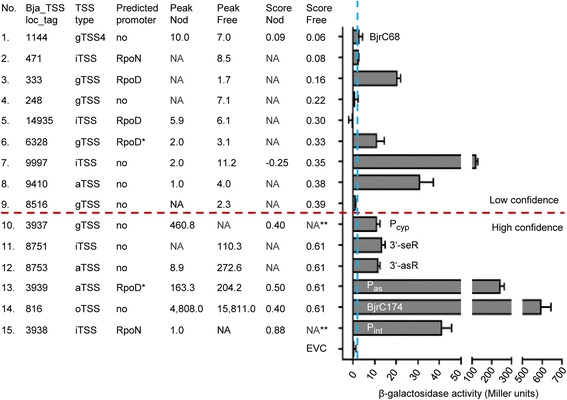
Promoter activities of TSS upstream regions. Beta-galactosidase activities of *B. japonicum* USDA 110 cells harboring plasmids with transcriptional *lacZYA* reporter fusions measured in exponentially growing cultures. Fifteen TSSs with scores between 0.06 and 0.88 (*ordered according to increasing score from top to bottom*) were tested. The number of each tested TSS (No.), its Bja_TSS_locus tag, peak height (*Peak*) and score in Free and Nod as well as the presence of a mapped promoter in its upstream region are indicated. NA, not applicable (indicates that the TSS was not scored under the respective condition; see Additional file 3: Table S3); no, promoter was not mapped upstream of the respective TSS; *, promoter was verified by mutagenesis (see Fig. 4d and Fig. 8); **, the TSS was validated in Free although it was scored only in Nod. The dashed blue vertical line separates the extremely low beta-galactosidase activity values indicating no promoter activity (and thus failed validation of five TSSs) from the higher beta-galactodsidase activity values validating ten TSSs. The dashed red horizontal line separates TSSs with scores below 0.4 belonging to the class of low confidence from the high-confidence TSSs with scores above the cut-off (see also Figs. 6 and 7 below). BjrC68 and BjrC174, previously analyzed sRNAs [16]. For P_cyp_, P_int_, P_as_, 3′-seR and 3′-asR, see Fig. 9 below

Out of all TSSs, 33 % are preceded by putative promoters identified in this study. This fraction ranges from 40 % for gTSSs or aTSSs to 14.5 % for iTSSs. This suggests that despite the stronger stringency for scoring iTSSs compared to gTSS and aTSS scoring, and despite the observation that most iTSSs were mapped in genes for which no gTSS was detected (Additional file 1: Table S2), many of the mapped iTSSs may not represent genuine TSSs and should be considered more cautiously than other TSS categories. To address this point, we tested the upstream regions of five iTSSs (no. 2, 5, 7, 11 and 15 in Fig. 5) for promoter activities and verified three of them (no. 7, 11 and 15): Bja_TSS_9997 (no. 7) and Bja_TSS_8751 (no. 11) without mapped promoters, and Bja_TSS_3938 (no. 15) with a mapped promoter. These results demonstrate the existence of real TSSs among the iTSSs without mapped promoters.

Our *de novo* promoter motif detection algorithm allows fast and sensitive promoter prediction with very large sequence and transcriptomics data sets. The algorithm, however, did not detect conserved motifs other than the typical RpoD- and RpoN-like motifs, although upstream of 67 % of all mapped TSSs neither of these two promoter types were identified. This may be due to three major reasons: (1) our tool is targeted to detect only highly abundant motifs; (2) unknown motifs are not sufficiently conserved to be detected by the algorithm; (3) to avoid false positives we have chosen a too high threshold for promoter identification. Nevertheless, our approach can be applied for global detection and mapping of promoters, as is shown by our prediction of RpoN-like promoters upstream of well-known RpoN-dependent genes [33]. Notably, the RpoN-promoter of the symbiotically important *fdxN* (bsr1739) gene, which was originally described as an unconventional but functional RpoN-dependent promoter [33], was correctly predicted by our algorithm. Its score of 2.9 belongs to the highest scores together with those of the RpoN-dependent genes *nifD* (score 3.7), *nifB* (score 3.2), *nifH* (score 3.8), *groESL*_*3*_ (score 3.9), *fixA* (score 2.9) and the *iscN* homolog blr1755 (score 3.5); (Additional file 8: Table S9) [33, 67, 68]. This indicates that the genome-wide map of RpoN-like promoter motifs is a reliable tool for further exploring the regulatory scope of RpoN.

### Experimental TSS validation and definition of a TSS class of lower confidence

To validate our TSS mapping, we decided to test experimentally TSSs with low scores. All 35 TSSs that were previously identified by others and mapped by our SVM method have scores between 3.1 (the TSS of *fixN* in Nod) and 0.4 (e.g., the TSSs of *fixA* in Nod; Additional file 3: Table S4). Further, the TSS of *rpoH*_*2*_ had the lowest score (0.6) of a previously determined TSS mapped only in free-living condition (Additional file 3: Table S4). Therefore we focused our validation experiments (based on promoter activity measurements in Free, see Methods) on TSSs with maximal scores of 0.6, with emphasis on TSSs with low peak heights and/or without mapped promoters.

The results for the 15 TSSs tested in this work are summarized in Fig. 5. We confirmed promoter activities upstream of 10 TSSs (including the promoter P_as_ shown in Fig. 4d). Six of the 10 validated TSSs lack mapped promoters (TSS no. 7, 8, 10, 11, 12 and 14 in Fig. 5), and thus their promoters do not match any of the consensus sequences shown in Fig. 4. The highest promoter activity for a TSSs without a mapped promoter was measured for no. 14, an oTSS belonging to the previously described abundant sRNA BjrC174 [16], followed by no. 7, an iTSS located in the middle of bll5007 (a gene of unknown function with proteomic evidence).

**Fig. 6:**
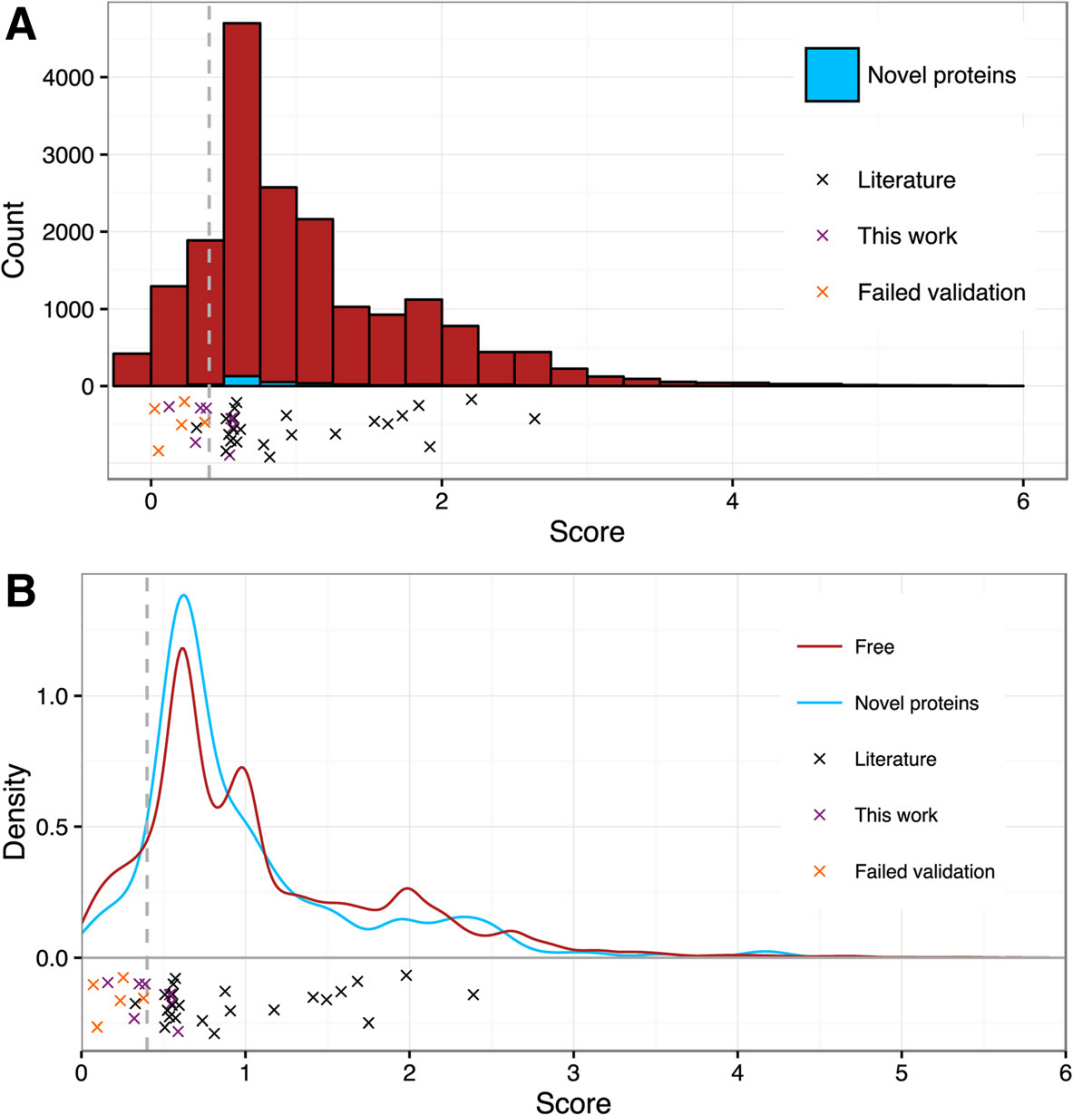
TSS score distribution in Free. Red, all TSSs mapped in Free; blue, TSSs (gTSS_ORF) of novel proteins (novel ORF or novel start site) mapped in Free; black crosses, literature TSSs; violet crosses, TSSs validated in this work; orange crosses, TSSs with failed validation; vertical dashed line at 0.4, score cut-off. **a** Histogram of score distribution. **b** Density plot of score distribution. The density plot allows us to compare score distributions of TSSs of novel proteins to the score distribution of all TSSs, despite considerable difference in number of respective TSSs. A negative score means that the TSS was not scored in one of the conditions (see also Additional file 3: Table S3)

**Fig. 7:**
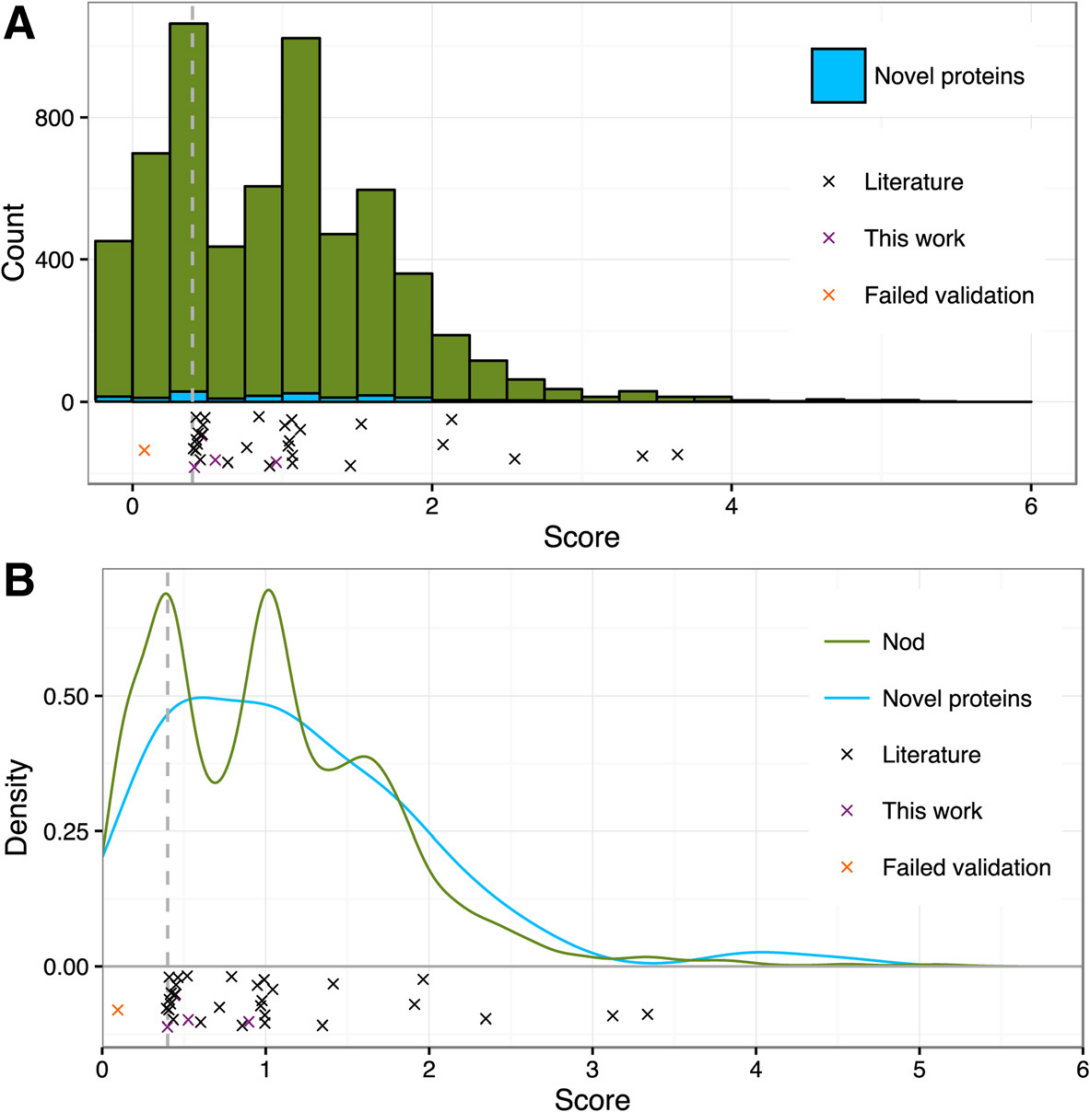
TSS score distribution in Nod. **a** Histogram of score distribution. **b** Density plot of score distribution. *Green*, TSSs mapped in Nod. For other descriptions see Fig. 6

Next we analyzed the score distribution of all mapped TSSs, the 45 experimentally validated ones and the five TSS that we failed to validate (Figs. 6 and 7; see also Fig. 5 and Additional file 3: Table S4). We also visualized the score distribution of 253 gTSSs assigned to 182 novel proteins which were identified by our proteomics approach providing independent experimental evidence for these TSSs. The histogram and density plots in Figs. 6 and 7 show that despite the relatively low number of proteomics-supported TSSs, their scores are distributed similarly to the scores of all mapped TSSs.

Based on the results summarized in Figs. 6 and 7, we set a score cut-off of 0.4 separating TSSs of high and low confidence. The scores of all five TSSs that we failed to validate were below 0.4, and all six TSSs with scores of 0.4 or higher, which we tested, were validated (see also Fig. 5). Further, as mentioned above, all TSSs validated previously by others have scores above this cut-off. Moreover, in symbiosis the majority of the TSSs of genes encoding novel proteins have scores above 0.4 (Fig. 7), and in free-living conditions approximately at this score value the density of TSSs of novel proteins starts to be higher than the density of all TSSs (Fig. 6). Thus we labeled all mapped TSSs with scores below 0.4 as “a class of lower confidence TSSs” (Additional file 3: Table S3). It is important to note that this class comprises some real TSSs: we validated four out of the nine tested TSSs with scores below 0.4 (Fig. 5) and 9 % of the TSSs of genes encoding novel proteins have scores below 0.4. To the TSSs above this cut-off belong 86 % of all mapped TSSs: 90 % of the gTSSs, 82 % of the iTSSs, 86 % of the aTSS and 86 % of the oTSSs (Additional file 10: Table S12).

### Analysis of TSSs preceded by weak promoters

**Fig. 8:**
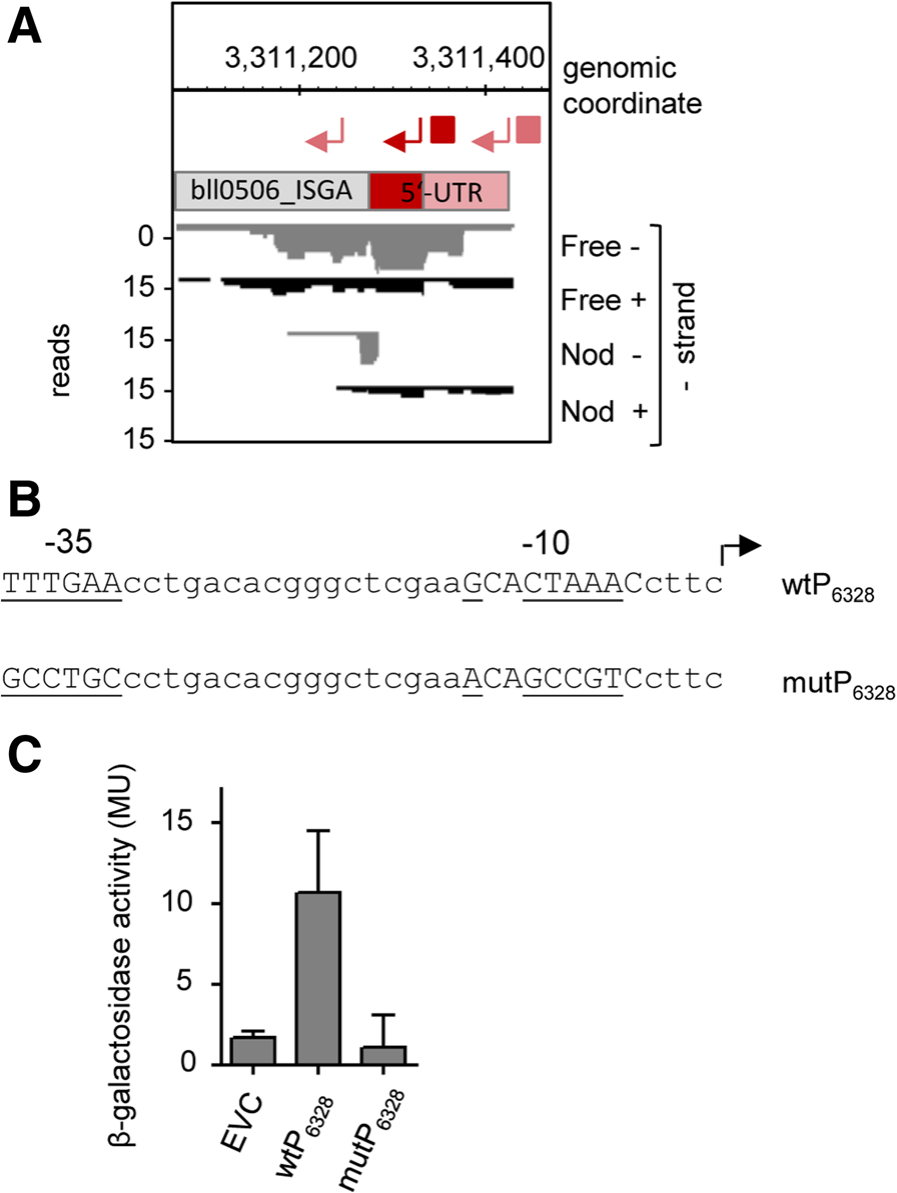
Validation of a TSS and a promoter of the newly annotated and proteomics-supported gene bll0506_ISGA. **a** The bll0506_ISGA locus with cDNA reads and mapped TSSs (*red flexed arrows*) and promoters (*red squares*). The analyzed TSS Bja_TSS_6328 with a low peak height and a low-score (no. 6 in Fig. 5), and its RpoD-like promoter are in dark red; non-analyzed TSSs and promoter are in light red. The bll0506_ISGA mRNA is shown as a bar with differently colored regions: grey, the annotated bll0506 ORF; dark red, the here verified 5′-UTR; light red, a proposed upstream part of a longer 5′-UTR. **b** The wild type sequence upstream of Bja_TSS_6328 containing the RpoD-like promoter (wtP_6328_) and the mutated version mutP_6328_. Bases subjected to mutagenesis are underlined. For other descriptions see the legend of Fig. 4d. **c** Beta-galactosidase activity measurements in *B. japonicum.* The wild-type and mutated versions of the 41 nt-region upstream of Bja_TSS_6328 (see panel **b**) were transcriptionally fused to the *lac* operon. Shown are results from three independent experiments with technical duplicates and error bars depicting the standard deviation

Among the TSSs with the lowest promoter activities (TSSs no. 6, 10, 11 and 12), only TSS no. 6 has a mapped promoter (Fig. 5). We validated this promoter by mutagenesis of the predicted –35 and –10 boxes, which completely abolished its activity (Fig. 8). Thus, we unambiguously verified Bja_TSS_6328, a TSS with a low peak height (3.1) and a low score (0.33), which belongs to the newly annotated gene bll0506_ISGA, which was also identified by our proteogenomics approach.

**Fig. 9:**
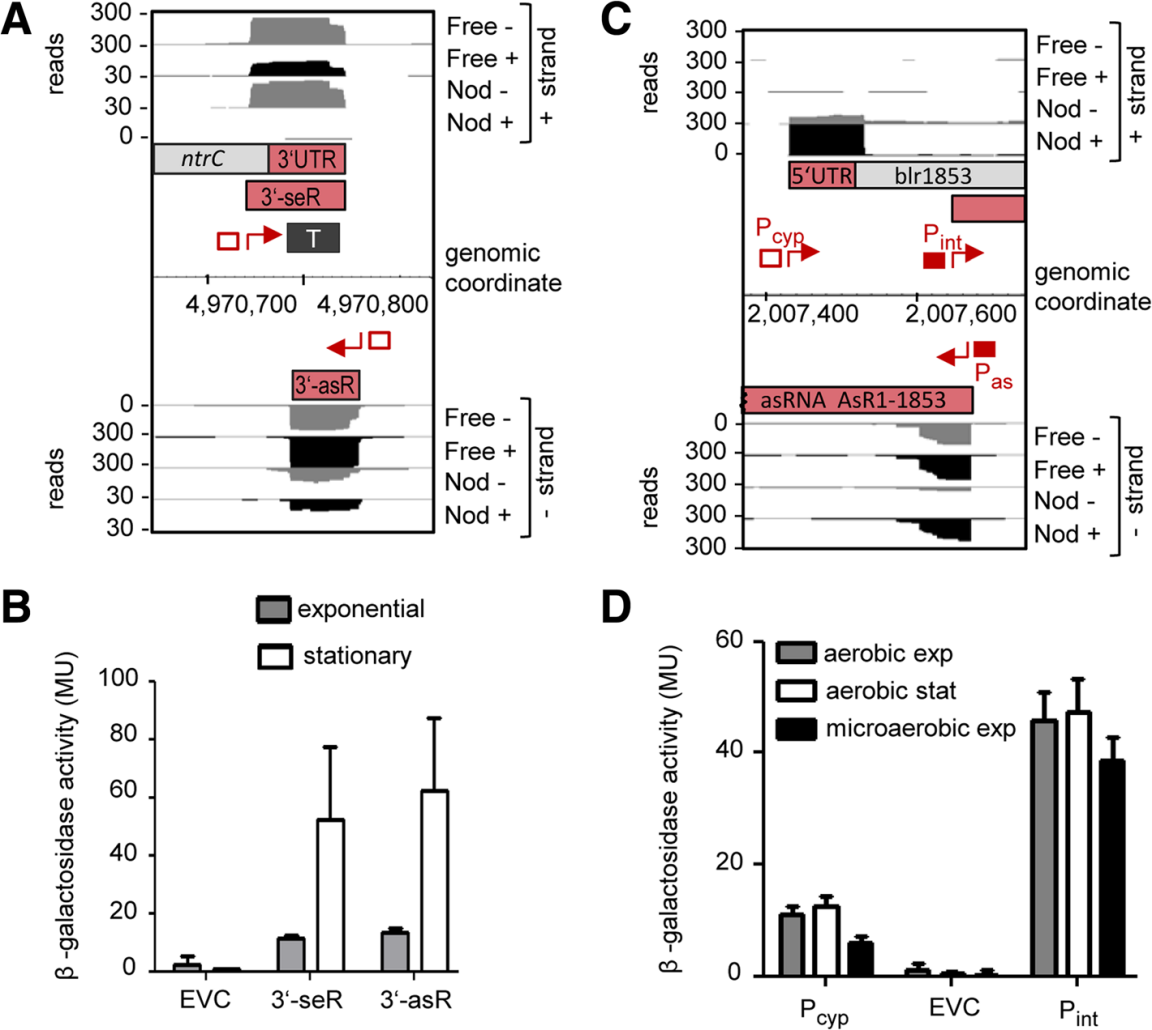
Promoter activities in the 3′-region of *ntrC* and upstream of blr1853 under different conditions. **a** The 3′-region of *ntrC* with two convergent TSSs without mapped promoters: Bja_TSS_8751 of the sRNA 3′-seR and Bja_TSS_8753 of the asRNA 3′-asR (designated no. 11, and 12 in Fig. 5). **b** The promoter activities of the regions upstream of the two TSSs shown in **a** were measured in the exponential and stationary growth phases. **c** The blr1853 locus with three TSSs: Bja_TSS_3937 preceded P_cyp_, Bja_TSS3938 preceded by P_int_ and Bja_TSS_3939 preceded by P_as_ (the respective numbers of these TSSs in Fig. 5 are no. 10, 15 and 13). **d** The activities of the promoters P_cyp_ and P_int_ were measured in aerobic cultures grown to the exponential and stationary growth phase, and in exponentially growing cultures under microoxic conditions. For the analysis of P_as_, see Fig. 4d. **a** and **c** show cDNA reads, mapped TSSs (*red flexed arrows*), mapped promoters (*red filled rectangles*), TSS upstream regions with promoter activities lacking mapped promoters (*red empty rectangles*), a mapped terminator (*dark grey rectangle marked with T*), mRNAs (bars with differently colored regions – light grey and red depicting annotated ORFs and UTRs, respectively) and new transcripts (red bars representing internal sense RNAs or as RNAs). **b** and **d** show the results from beta-galactosidase measurements in *B. japonicum*. Respective 200 nt TSS upstream regions were cloned in front of the *lac*-operon. Shown are results from three independent experiments with technical duplicates and error bars depicting the standard deviation

To provide additional support for the TSSs no. 10, 11 and 12 (Fig. 5), we decided to validate them also under additional conditions. The TSSs no. 11 and 12 correspond to a sense and an antisense RNAs (3′-seR and 3′-asR), which overlap with the 3′-UTR of *ntrC* (Fig. 9a), a gene encoding a regulator of nitrogen metabolism [69]. Figure 9b shows that their promoter activities are higher in the stationary than in the exponential growth phase, suggesting a growth-stage-specific regulation which may also influence *ntrC* expression. TSS no. 10 is the gTSS of blr1853, a gene encoding a cytochrome P450 protein (CYP) and known to be highly expressed in nodules (Fig. 9c; [13, 70]). The promoter activity (P_cyp_) was similar in aerobic exponential and stationary growth phase cultures, and was slightly lower under microaerobiosis (Fig. 9d), a condition which is known to induce many symbiosis-relevant genes [3, 67, 71]. The last result is consistent with previous microarray data and suggests that blr1853 is specifically induced in symbiosis [13].

Between the TSSs preceded by P_cyp_ (no. 10, described above) and P_as_ (no. 13, verified in Fig. 4d) we mapped an additional iTSS with a promoter P_int_ (no. 15 in Fig. 5; Fig. 9c), the activity of which was similar in the exponential and stationary growth phase and under microaerobiosis (Fig. 9d). The convergent arrangement of P_int_ and P_as_ suggests that they may influence each other and/or the expression of blr1853 [40]. To address the role of blr1853 and its asRNA AsR1-blr1853 (Fig. 9c), we overproduced the asRNA from a plasmid in the sense and antisense direction, both in free-living cells and in bacteroids, but this had no effect on the blr1853 mRNA level (see Additional file 2: Figure S6). It is noteworthy that the TSSs no. 10 and 15 preceded by P_cyp_ and P_int_ were validated in free living conditions, but were scored only in symbiosis (Fig. 5 and 9d).

In summary, we were able to map a set of TSSs with low scores and low peak heights and to predict corresponding promoters. Thus, our TSS map enables detection not only of highly expressed transcripts, but also of low-abundant ones.

## Conclusions

We analyzed the primary transcriptome of the soybean symbiont *B. japonicum* USDA 110 grown under free-living and symbiotic conditions, and provide the first genome-wide TSS and promoter maps for this bacterium. TSS recognition was performed with a specialized tool based on machine learning which enabled fast and sensitive global mapping of 14,360 TSSs in free-living bacteria and 4329 TSSs in bacteroids within the large *B. japonicum* genome. The TSS map served as a basis for *de novo* prediction of promoter motifs with similarity to RpoD- and RpoN-dependent promoters by a new algorithm. The algorithm is publicly available and will be useful for *de novo* prediction of bacterial promoters. Combining the global TSS map with a proteogenomics approach proved to be a powerful solution and led to an extension of the repertoire of protein-coding genes, providing evidence for 107 new proteins and identifying different N-termini for 178 proteins compared to the existing annotation. The score distribution of previously mapped TSSs, TSSs validated in this study and TSSs of new protein genes allowed us to define a score threshold that flags a lower confidence class of TSSs. This lower confidence class contains some functional TSSs of weakly expressed genes. Mapped TSSs and promoters were included in re-annotation files along with the proteomics evidence and predicted terminators and operons. Our updated and extended annotation is a valuable resource for both future systems biology studies or for in-depth analyses of specific genes and their regulation in *B. japonicum* and related bacteria.

## Methods

### Cultivation methods

The rhizobial strain *B. japonicum* 110*spc*4 [72], a spectinomycin-resistant derivative of *B. japonicum* USDA 110, was either grown in liquid cultures in PSY medium [71] with spectinomycin (100 μg ml^−1^), or in symbiosis with soybean plants as described [13]. Liquid cultures were cultivated aerobically or microaerobically, under a gas atmosphere that initially contained 2 % oxygen. Cells were harvested in the exponential or stationary growth phase as indicated. *E. coli* was cultivated in LB medium. Plasmids conferring resistance to bacterial strains are listed in Additional file 11: Table S13.

### RNA isolation and dRNA-seq

Total RNA from free-living cells in the exponential growth phase (OD_600_ of 0.4 to 0.6) or stationary phase (OD_600_ of 1.2) as well as total RNA from soybean nodules and from uninfected soybean roots was isolated with hot-phenol [13]. For Differential RNA sequencing (dRNA-seq), RNA isolated from 3 independent cultures harvested at OD_600_ = 0.5 was pooled. dRNA-seq of total RNA from nodules (Nod) and from cultures (Free), as well as subsequent read mapping to the *B. japonicum* USDA 110 (NC_004463) and soybean (*G. max*) genomes using the READempion pipeline (pre 0.1) [73] *segemehl* 0.1.3 software [74] was performed as previously described [16, 19]. For each library (Free –/+ TEX and Nod –/+ TEX) graphs representing the number of mapped reads per nucleotide were calculated and visualized using the Integrated Genome Browser software from Affymetrix as described [75].

### Annotation of TSSs

To automate TSS detection and retain human expertise, we designed a machine-learning approach for dRNA-seq data analysis.

#### Calculating peaks of salience function

For each experimental condition we selected peaks of (+) and (–) libraries, which were separated by 0-2 nt. Coordinate of a peak (and corresponding TSS) is the coordinate of the peak in the (+) library. Peaks were defined as sharp jumps in read coverage indicated by local maxima of the salience function (see Figure S1 in Additional file 2), and this is how the term “peak” is used throughout the article. To calculate the salience function, each library was preprocessed using the following procedure. First the following convolution values are calculated:

$$ Y\left[j\right]={\displaystyle {\sum}_if}\left[i\right]{H}_m\left[\left|i-j\right|\right], $$

where *H*_*m*_ is defined as:

$$ Hm=\left\{\begin{array}{cc}\hfill 1/m,\hfill & \hfill -m\le x\le 0,\hfill \\ {}\hfill \begin{array}{l}-1/m,\\ {}0,\end{array}\hfill & \hfill \begin{array}{l}0<x\le m,\\ {} otherwise.\end{array}\hfill \end{array}\right. $$

Here *f[i]* is the read count; *m*, the convolution step; |*i*−*j*| corresponds to the distance between coordinates *i* and *j*, positive for the upstream and negative for the downstream coordinate. The convolution step *m* equals to 10 bp.

In order to minimize the number of false peaks, only local maxima at each bp coordinate were retained, removing all but the largest-magnitude peaks within a 5 bp window.

#### Machine learning

To sort out the peaks corresponding to non-primary transcripts, we performed an SVM-based machine learning using SVMTorch [76] with the Radial Basis Function (RBF) kernel. Putative TSSs, which had scored positively in the preliminary scoring, were manually assessed in two genomic regions with coordinates 0 to 130 kb and 1681 to 1920 kb, the latter being the beginning of the symbiotic region. Totally 220 putative TSSs were assessed, with 164 peaks detected in both conditions: 156 out of 202 peaks in Free were evaluated as true TSSs and assigned to the positive set, while the remaining 46 peaks were assigned to the negative set; similarly, 135 out of 182 peaks in Nod were assigned to the positive set and 47 were assigned to the negative set (see also Additional file 5).

The machine learning was performed separately for Free and Nod. To compute the support vectors, the following parameters were selected: (1) height of peak in the (+) library, (2) height of peak in the (–) library, (3) ratio of the (+) and (–) peaks, (4) average read coverage within the 30 bp interval in the (+) library, (5) average read coverage within the 30 bp interval in the (–) library, (6) salience function value for the 30 bp radius in the (+) library, (7) salience function value for the 30 bp radius in the (–) library, (8) number of read starts within the 4 bp radius in the (+) library, (9) number of read starts within the 4 bp radius in the (–) library and (10) distance between peaks in the (+) and (–) libraries. The parameters were selected so as to mimic factors influencing the expert judgment in the manual annotation of TSSs, reflecting expression patterns at different scales.

For further analysis, peaks were considered to be present in both experimental conditions (Nod and Free), if they were separated by less than 3 bp. The joint coordinate of such merged TSS was calculated as the average of two, and rounded to the nearest whole number. If the two coordinates were equally close to the average, the 3′-coordinate was considered. The peak was retained as a TSS if it scored positively in SVM in at least one of the two experimental conditions. For the distribution of TSS candidates and retained TSSs in Nod and Free, see Additional file 2: Figure S2.

#### Additional filtering for iTSSs

For the expressed genes it can occur that processed (non-primary) transcripts are not fully digested by TEX and thus have peaks classified as iTSS, although they probably are processed 5′-ends. Thus, if a TSS mapped inside an annotated gene possessing at least one gTSS, the iTSS was retained only if its (+)-to-(–) peak ratio exceeded the ratio for the gTSS.

### Genome re-annotation

An updated annotation of the *B. japonicum* USDA 110 genome was generated in July 2013 by submitting its genomic sequence to the Ergatis pipeline of Integrated Services of Genomics Analysis (ISGA). In comparison to the RefSeq annotation [4], the ISGA annotation improved the ratio of gene TSSs to internal TSSs (see Additional file 4). We preserved the original gene identifiers of the RefSeq annotation [4] and added, when appropriate, ISGA numbers of newly predicted genes, keeping the designation of locus tags used [4]. We included additional features to our annotation file, namely the TSSs and promoters mapped in this study, and predicted operons (based on the ProOpDB database; [77]) and Rho-independent terminators (mapped using the tools ARNold, WebGesterGB and TransTermHP; [78–80]). For more details see Additional file 4.

### Proteomics evidence for longer, shorter and novel ORFs

Existing proteomics data of *B. japonicum* 110 grown under free-living conditions (rich (PSY) and minimal medium, [56], and in symbiosis with soybean (*G. max,* [15]), cowpea (*V. unguiculata*) or siratro (*M. atropurpureum*) [57] was re-analyzed as follows: fragment ion mass spectra were searched with MS-GF+ (MS-GFDB v9979, [81]) against a protein database containing sequences of 8317 *B. japonicum* USDA 110 proteins, 2857 shorter ORFs and 194 longer ORFs, 1391 newly predicted ORFs, 5894 protein sequences generated by *in-silico* translation starting from 593 iTSS with strong dRNA-seq evidence (up to 200 nt downstream), and 256 common contaminants (e.g., human keratin, trypsin). In total, the protein database contained 18,909 protein sequences. Spectra were searched for a match to fully-tryptic and semi-tryptic peptides with a mass tolerance of 25 ppm. Carbamidomethylation was set as fixed modification for all cysteines, while oxidation of methionines was considered as optional modification. Based on the target-decoy search strategy a stringent score cutoff was determined that resulted in an estimated FDR of 0.1 % at the peptide spectrum match (PSM) level. PSMs above this cutoff were subjected to a PeptideClassifier analysis [82] and only peptides that unambiguously identify one protein, or that imply a longer or shorter from of an annotated protein (extending the concept of Gerster et al. [83]), were considered. We furthermore required at least 3 independent spectra for a protein identification as described [84], which resulted in a total of 4090 identified protein groups at an estimated protein level FDR below 1 % (0.9 %).

### *De novo* prediction of promoters from genome-wide samples

At the first step, we aimed to find *patterns*, that is, pairs of 6-mers separated by a spacer, which are overrepresented in a given sample of sequences. Patterns were scored using a flexible scheme that allows for mismatches in sequence and deviations in position of both 6-mers. We selected 6-mers that occur together more frequently than expected given their individual frequencies by scanning all possible 6-mers at all positions. As the average GC-content of the genome is 0.64, GC rich 6-mers would occur more frequently by pure chance. To account for that, we normalized the frequencies of patterns by their GC-content. At the second step, *motifs*, that is, distinct clusters of overrepresented patterns, were identified, followed by construction of a PWM representation for each motif. Finally, at the third step, we identified the highest scoring pattern for each motif in each TSS upstream region, and used these scores to select the relevant motifs. For more details on promoter prediction see Additional file 4.

### RT-PCR and qRT-PCR analyses

We used RT-PCR and qRT-PCR in order to estimate the approximate length of the asRNA AsR1 and to compare the steady state levels of AsR1 and of the complementary mRNA blr1853. Used oligonucleotides are listed in Additional file 11: Table S14. For more details, see Additional file 4.

### Cloning procedures

Standard cloning methods were used [85]. Plasmids for biparental conjugational transfer from *E. coli* S17-1 to *B. japonicum* USDA 110 were constructed as previously described [86] (see also Additional file 4). Routinely, for promoter verification PCR products corresponding to 200 nt regions located upstream of a mapped TSS were cloned upstream of the promoterless *lacZYA* operon as described in Additional file 4. Exceptions were the promoter regions analyzed by mutagenesis: to clone the 63 nt region upstream of Bja_TSS_3939 and the 41 nt region upstream of Bja_TSS_6328, suitable complementary oligonucleotides with integrated restriction sites were annealed and cloned upstream of *lacZYA*. Further, to validate the predicted P_int_, the region between Bja_TSS_3937 and Bja_TSS_3938 was cloned. The resulting reporter fusions were transferred into *B. japonicum* and beta-galactosidase activity of cells grown in liquid cultures was measured.

### Promoter activity measurements

Beta-galactosidase assays were performed as described previously [87] with cells from 1 ml culture grown aerobically or under microaerobiosis and harvested in the exponential or stationary phase.

### Calculation of distributions

Graphical representation of distributions shown in Fig. 2c, Fig. 6b and Fig. 7b were prepared with R ggplot2 [88] with standard settings for box plot and standard kernel settings for density plot.

### Ethics approval and consent to participate

Not applicable

### Consent for publication

Not applicable

### Availability of data and material

The RNA-seq data set supporting the conclusions of this article is available in the [NCBI’s Gene Expression Omnibus] repository [GEO Series accession number GSE69059 and https://www.ncbi.nlm.nih.gov/geo/query/acc.cgi?acc=GSE69059] [89]. The promoter prediction tool is available [https://github.com/mimakaev/promoter_mapper]. All other datasets supporting the conclusions of this article are included within the article and its Additional files.

## Additional files

Additional file 1: Table S1 - Read mapping statistics; Table S2 - Numbers of annotated genes and operons associated with different TSSs. (PDF 87 kb) Additional file 2: Supplementary figures. (PDF 712 kb) Additional file 3: Table S3 listing all mapped TSSs; Table S4 listing previously published TSSs compared to our TSS mapping. (XLSX 1436 kb) Additional file 4: Supplementary methods. (PDF 1355 kb) Additional file 5: Annotation file gff. (ZIP 3448 kb) Additional file 6: Annotation file gbk. (ZIP 7457 kb) Additional file 7: Table S5 - List of all detected proteins; Table S6 - Comparison of our proteomics data to ref. [61]. (XLS 3565 kb) Additional file 8: Table S7 - Predicted RpoD-like promoters; Table S8 - Predicted promoters with similarity to promoters found upstream of leaderless transcripts; Table S9 - Predicted RpoN-like promoters. (XLS 2957 kb) Additional file 9: Table S10 - gTSSs with coinciding RpoD-like and RpoN-like promoters; Table S11 - gTSSs with multiple promoters. (XLS 138 kb) Additional file 10: Table S12 - Percentages of different types of TSSs above score cut-offs. (XLSX 11 kb) Additional file 11: Table S13 - Plasmids used in this work; Table S14 – Oligonucleotides used in this work. (XLSX 15 kb)

## Abbreviations

asRNA: antisense RNA
EVC: empty vector control strain
Free: free-living state
gbk: gene bank sequence format
gff: generic feature format
IGR: intergenic region
kb: kilobase
Mb: megabase
Nod: nodule
nt: nucleotide
PCA: principle component analysis
qRT-PCR: quantitative reverse transcriptase-polymerase chain reaction
*rrn*: ribosomal RNA operon
rRNA: ribosomal RNA
RT-PCR: reverse transcriptase-polymerase chain reaction
sRNA: small RNA
SVM: support vector machine
TEX: terminal exonuclease
TSS: transcriptional start site
WT: wild type
