# Supplementary material for: Genome-wide transcription start site mapping of Bradyrhizobium japonicum grown free-living or in symbiosis – a rich resource to identify new transcripts, proteins and to study gene regulation
Source: BMC Genomics. 2016 Apr 23;17:302. doi: 10.1186/s12864-016-2602-9 (PMC4842269; doi:10.1186/s12864-016-2602-9)
Supplement: Additional file 1: — Table S1 - Read mapping statistics; Table S2 - Numbers of annotated genes and operons associated with different TSSs. (PDF 87 kb) [file 12864_2016_2602_MOESM1_ESM.pdf]

Additional File 1: Supplementary Tables S1 and S2.

	Condition (TEX treatment)			
**Read Statistics**	**Free (–)**	**Free (+)**	**Nod (–)**	**Nod (+)**
Number of raw reads	2,562,586	872,577	3,637,968	1,810,278
Total number of mapped reads	2,447,113	822,112	3,000,241	1,685,581
Fraction of reads mapped to *B. japonicum* USDA 110 genome	95,62 %	91,17 %	13,68 %	22,73 %
Fraction of reads mapped to *Glycine max* genome	0,35 %	0,09%	70,51 %	72,27 %
Fraction of non-mapped reads	3,47 %	3,73%	15,70 %	4,72 %
Number of reads mapped to *B. japonicum* USDA 110 genome	2,432,900	783,084	487,925	405,948
Number of reads mapped to *B. japonicum* rRNA	1,439,054	153,725	336,998	64,278
Number of reads of *B.japonicum* after filtering reads mapped to rRNA	993,846	629,359	150,927	341,670
Fraction *B.japonicum* of reads mapped to rRNA	59.002 %	19.163 %	68.996 %	15.036 %

Supplementary Table S1. Read mapping statistics. Numbers of reads obtained by dRNA-seq and of reads mapped to the genome of *B. japonicum* USDA 110 (NC_004463) are shown (reads mapping to the *Glycine max* genome were not considered further). Free (–), reads of the RNA sample from exponentially growing, free- living cells, without TEX treatment; Free (+), reads of the RNA sample from exponentially growing, free- living cells, with TEX treatment; Nod (–, reads of the RNA sample from nodules, without TEX treatment; Nod (+), reads of the RNA sample from nodules, with TEX treatment.

**TSS category**	**No. of genes**	**No. of expressed genes**	**No. of operons (genes in these operons)**	**No. of expressed operons (genes in these operons)**
	9,245	4,214	6,352 (9,245)	3,324 (5,005)
iTSS	3,543	1,664	2,632 (4,921)	1,514 (2,931)
aTSS_5	1,095	295	1,095 (1,602)	388 (681)
aTSS_i	1,933	909	1,772 (3,336)	938 (1,851)
aTSS_3	782	419	782 (1,098)	419 (604)

Supplementary Table S2. Numbers of annotated genes and operons associated with different TSSs. Expressed genes or operons are genes or operons with mapped gene TSSs. No., number.
