# Supplementary material for: Genome-wide transcription start site mapping of Bradyrhizobium japonicum grown free-living or in symbiosis – a rich resource to identify new transcripts, proteins and to study gene regulation
Source: BMC Genomics. 2016 Apr 23;17:302. doi: 10.1186/s12864-016-2602-9 (PMC4842269; doi:10.1186/s12864-016-2602-9)
Supplement: Additional file 2: — Supplementary figures. (PDF 712 kb) [file 12864_2016_2602_MOESM2_ESM.pdf]

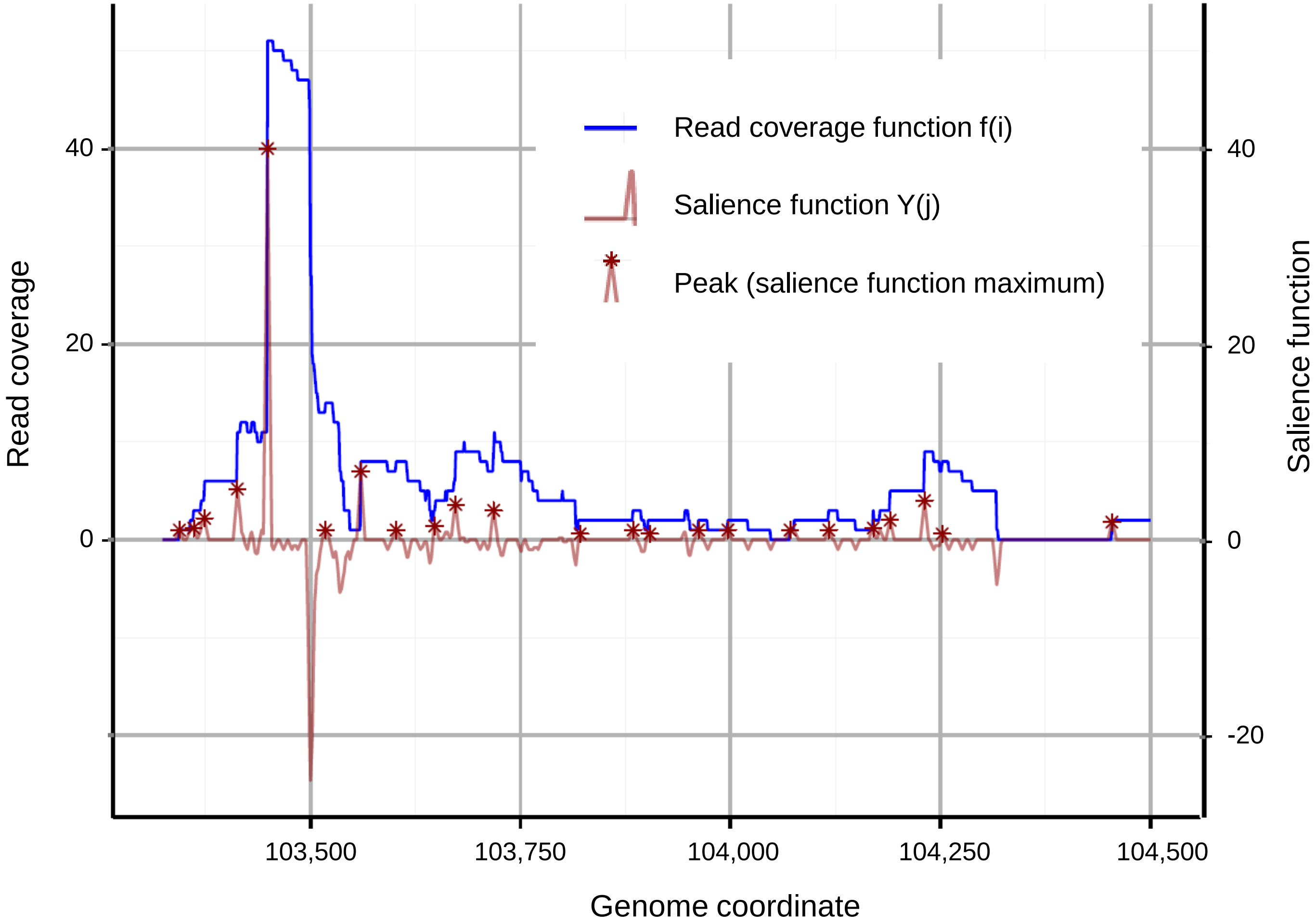

Supplementary Figure S1. Fragment of dRNA-seq Free(–)-library with local maxima of salience function termed "peaks". The function f(i) reflecting genome coverage by dRNA reads is shown in blue. The salience function Y(j), defined in the text (see Methods), is shown in red. The salience function characterizes changes in read coverage: it is positive when coverage increases and negative when coverage decreases. Red stars denote the peaks of the salience function, used as candidate TSSs.

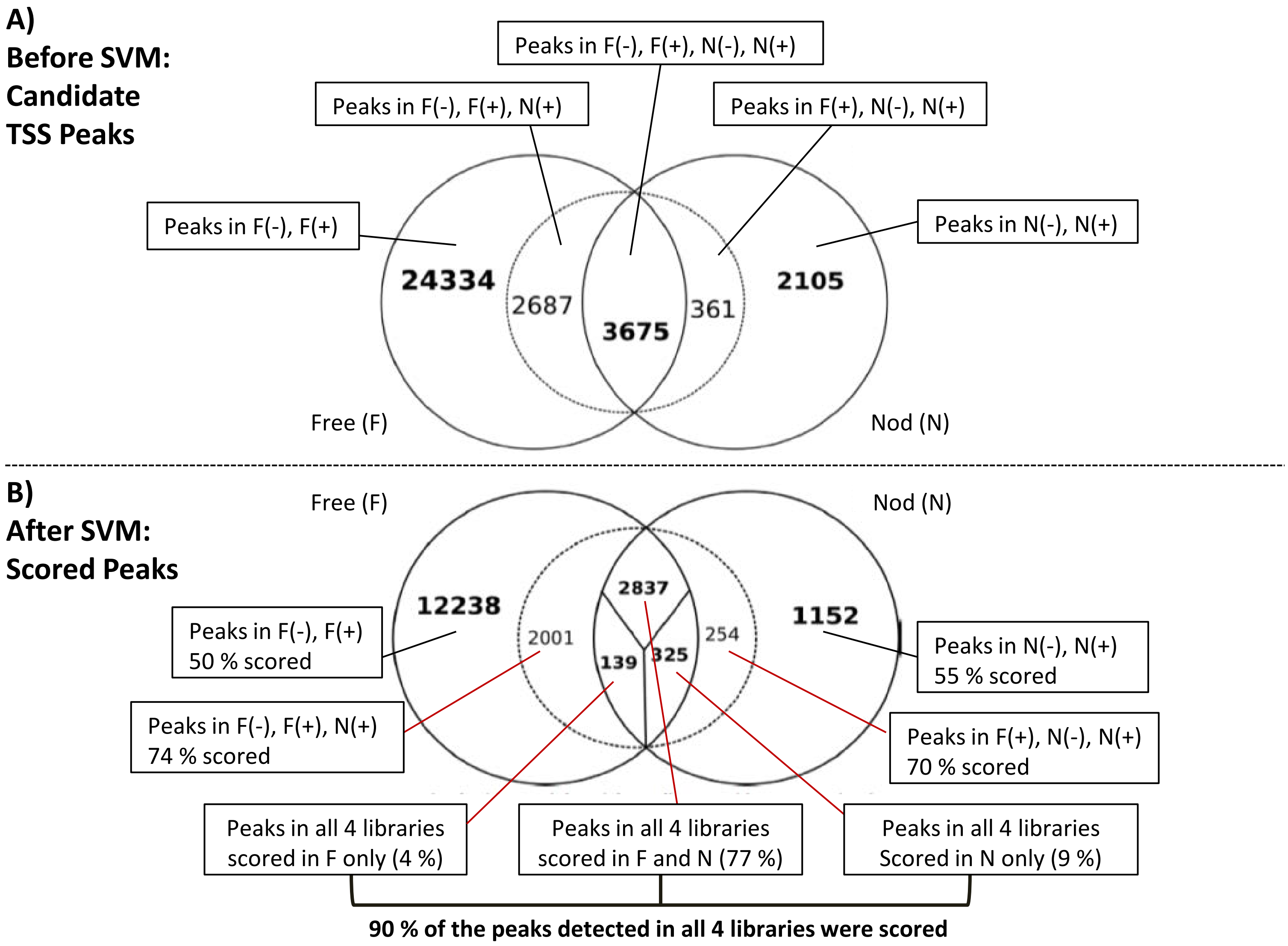

Supplementary Figure S2. Number of candidate TSSs in *B. japonicum* USDA 110 before (A) and after (B) SVM filtering. (**A**) Candidate TSSs detection before SVM. Candidate TSSs were defined as peaks with matching coordinates in the (+) and the (-) libraries under at least one of the experimental conditions (Free or Nod). Peaks within the dotted circle were detected in both libraries under one of the experimental conditions and in the (+) library only under the second condition. Peaks detected under both conditions in the (+) and (-) libraries are shown in the central ellipse. (**B**) TSSs detection after SVM filtering. Peaks scored positively in SVM in at least one environment were retained for further analysis. Peaks detected and scored positively in both environments are shown in the top sector of the central ellipse, and peaks scored positively in Free or Nod samples only are shown in the respective side sectors of the ellipse. Importantly, the ratio of scored peaks is higher for peaks within dotted circles (peaks with additional support from the second condition), compared to outside peaks (detected solely in one condition). As peaks detected as (+)-peaks in both conditions are more likely to be true TSS, this demonstrates that the scoring scheme performs well.

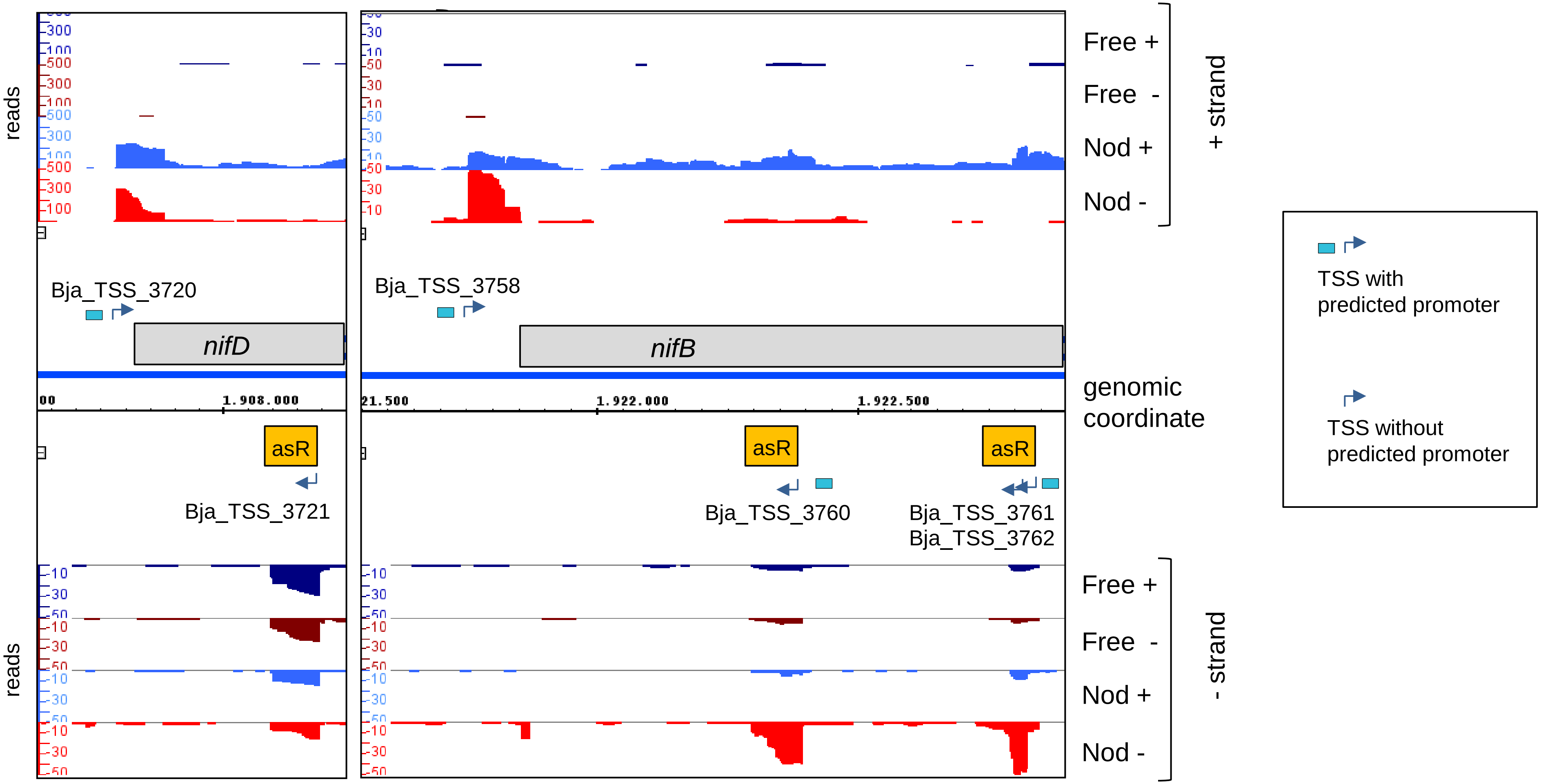

Supplementary Figure S3. Antisense RNAs transcribed opposite to *nifD* and *nifB* genes in *B. japonicum* USDA 110.

cDNA reads mapped to the 5'-region of the *nifD* and *nifB* loci. RNA was isolated from exponentially growing, free-living cells (Free) in liquid cultures and from nodules (Nod). RNA samples were treated (+) or not treated (-) with TEX. Gray bars, annotated transcripts; orange bars, new asRNAs. The orange bars cover the asRNA part, which was well detected by dRNA-seq. This does not exclude that the asRNAs are longer; their 3'-ends are not known. Shown are relevant, mapped TSSs (flexed arrows) and promoters (blue bars) . RpoN-dependent promoters were predicted upstream of the TSSs of *nifD* amd *nifB*, while promoters with similarity to RpoD-dependent promoters or no promoters were predicted upstream of the TSSs corresponding to the asRNAs. The RefSeq and ISGA annotations for *nifD* and *nifB* match perfectly. For more annotation details, see Additional files 5 and 6.

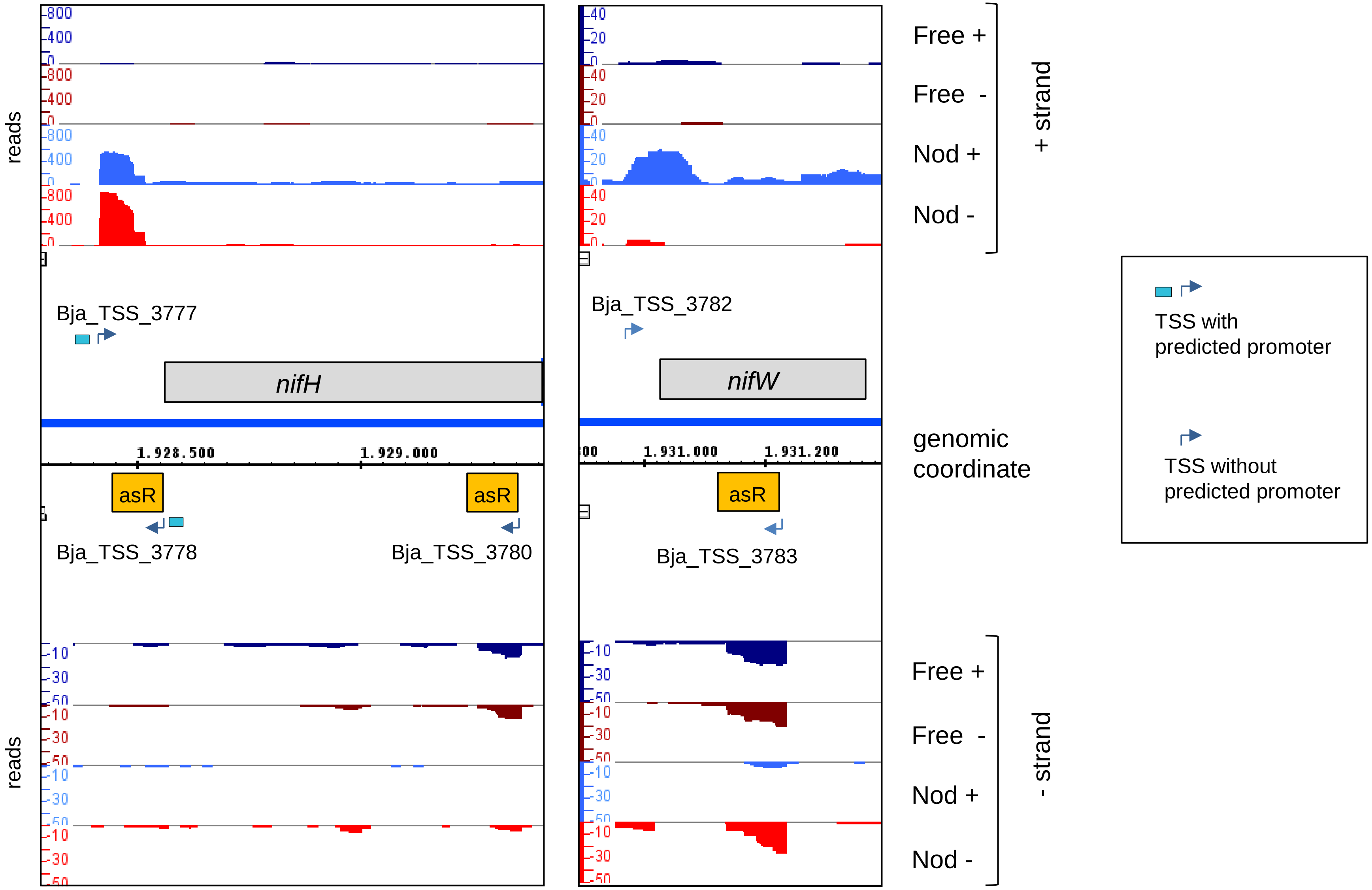

Supplementary Figure S4. Antisense RNAs transcribed opposite to *nifH* and *nifW* genes in *B. japonicum* USDA 110.

cDNA reads mapped to the 5'-region of the *nifH* and *nifW* loci. RpoN-dependent promoter was predicted upstream of the TSS of *nifH*; no promoter was predicted upstream of *nifW*; promoters with similarity to RpoD-dependent promoters or no promoters were predicted upstream of the TSSs corresponding to the asRNAs. The RefSeq and ISGA annotations for *nifH* and *nifW* match perfectly. For detailed description see the legend of the Supplementary Figure S3 on page 3. For more annotation details, see Additional files 5 and 6.

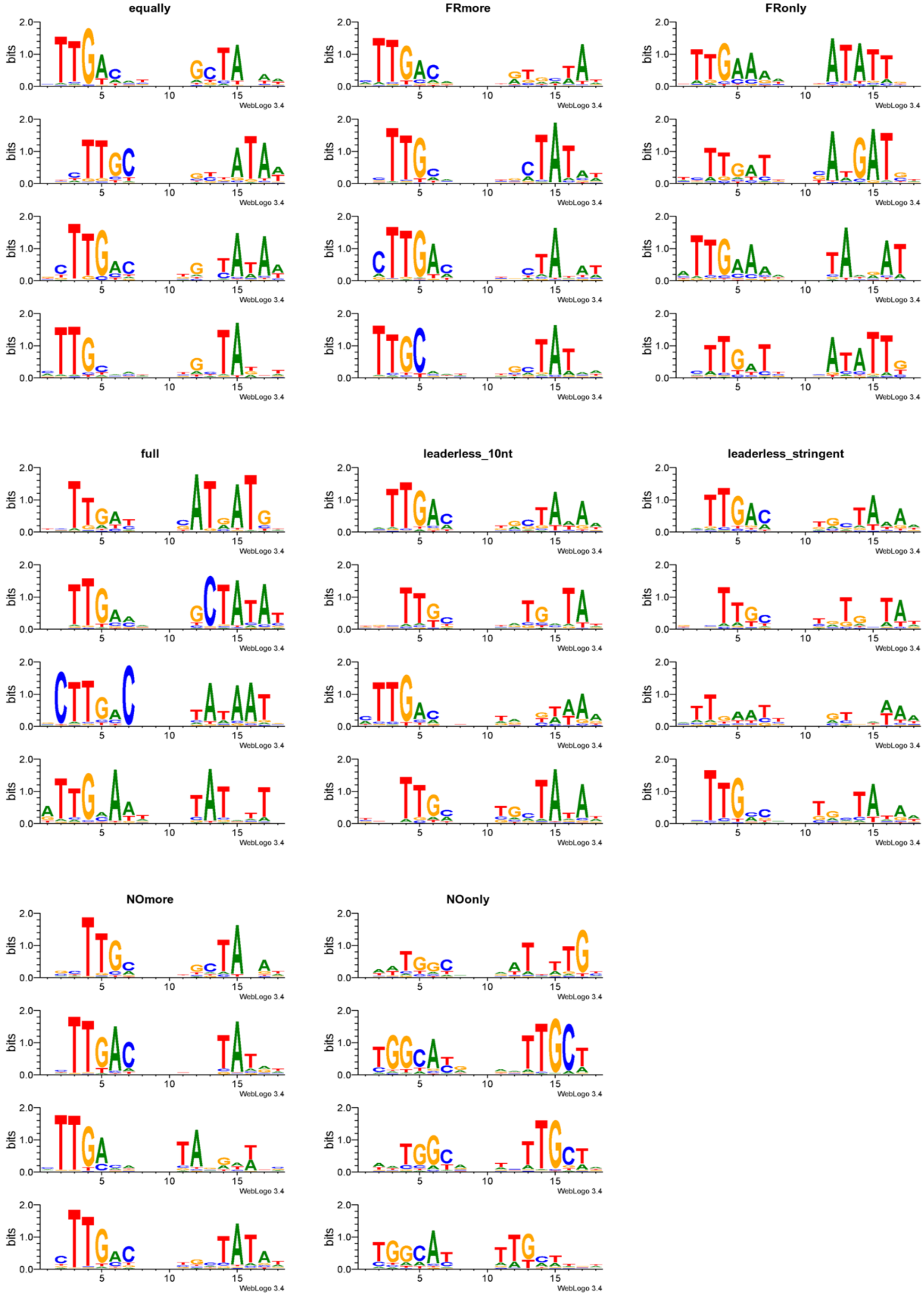

Supplementary Figure S5. PCA-derived logos for different classes of upstream regions. In an attempt to detect different motifs, we classified the mapped TSSs into five groups (classes): TSSs that are detected only in Nod (NOonly), have higher peaks (at least by 20 %; for the peak definition, see Methods) in Nod than in Free (NOmore), have peaks of similar height in Nod and Free (equally), have higher peaks in Free than in Nod (FRmore), or have peaks detected only in Free (FRonly). Additional classes: full, upstream regions of all mapped TSSs; leaderless_10nt, TSSs located up to 10 nt upstream of start codons; leaderless_stringent, TSSs located at the start codon. Shown are logos for the first principal components for all classes of upstream regions.

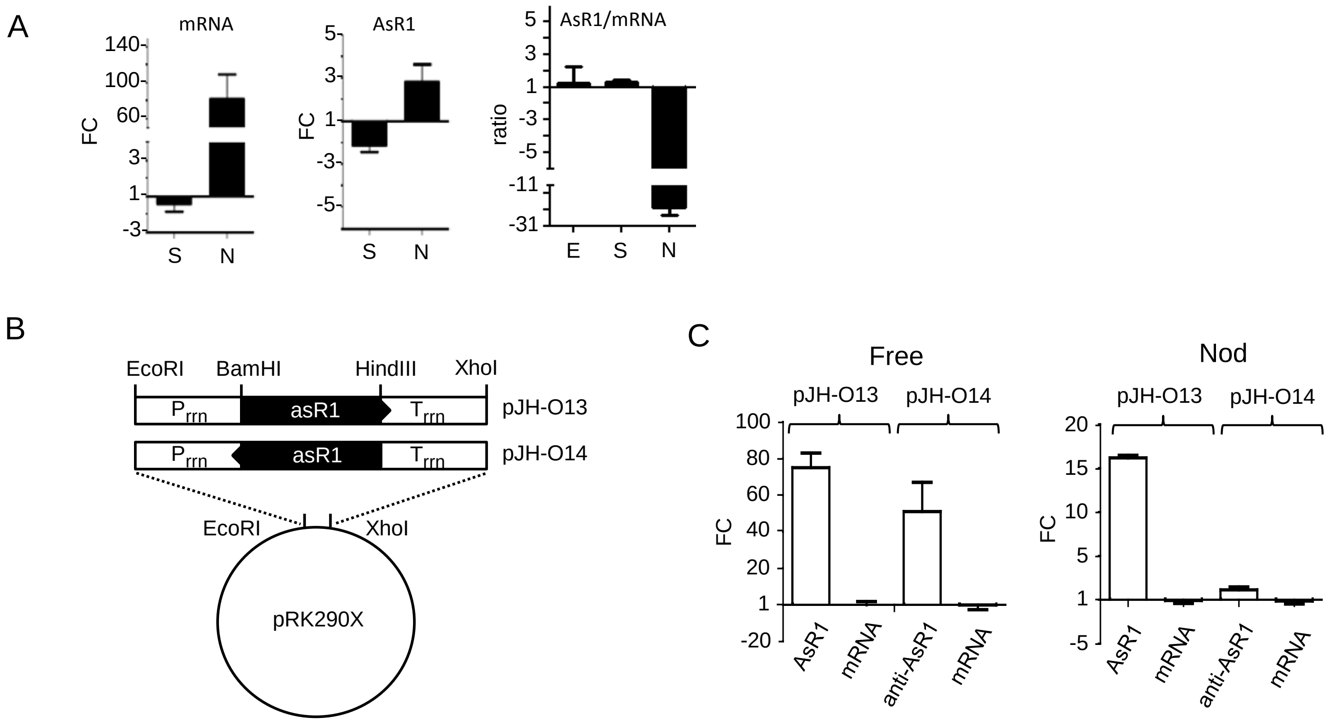

Supplementary Figure S6. Analysis of the mRNA blr1853 and its asRNA AsR1.

(A) Analysis of the the levels of the blr1853 mRNA and its asRNA AsR1 under three conditions: cells exponentially growing in liquid culture (E, corresponds to Free in dRNA-seq); cells in the stationary phase in liquid cultures (S); and bacteroids in nodules (N, corresponds to Nod). Fold change (FC) in the levels of the blr1853 mRNA (mRNA), AsR1, and in the AsR1 to mRNA ratio under the indicated conditions were determined by quantitative reverse transcriptase-PCR (qRT-PCR). In the left and middle panels, the levels in E were set to 1, and fold changes in S and N were calculated. This strand-specific qRT-PCR revealed that, relative to the exponential growth phase, the asRNA level varied slightly in stationary-phase cells and in bacteroids, while the blr1853 mRNA level was approximately 90-fold higher in nodules but did not change in the stationary phase . The low amount of AsR1 in comparison to the mRNA in nodules does not argue against a function in regulation of blr1853, since an asRNA, which is even 300-fold less abundant than the corresponding mRNA, can still significantly influence the level of the latter and impact the bacterial physiology (ref. 47 in the main text).

(B) Structure of plasmids pJH-O13 and pJH-O14 used for overproduction of AsR1 in sense and antisense orientation, respectively. P_{rrn}, ribosomal RNA operon (*rrn*) promoter, T_{rrn}, *rrn* terminator.

(C) Overproduction of AsR1 does not influence the level of blr1853 mRNA. Fold change (FC) in the levels of the mRNA, AsR1, and anti-AsR1 in liquid culture (Free) and under symbiosis (Nod) as determined by qRT-PCR. Levels in the EVC were set to 1, and fold changes in the strains containing either pJH-O13 or pJH-O14 were determined. The increase of the anti-AsR1 levels in symbiosis was hardly detectable due to the strong increase in the mRNA amount (see panel A). Shown are the results from two independent experiments with technical duplicates. Error bars depict the standard deviations.
