# Supplementary material for: Genome-wide transcription start site mapping of Bradyrhizobium japonicum grown free-living or in symbiosis – a rich resource to identify new transcripts, proteins and to study gene regulation
Source: BMC Genomics. 2016 Apr 23;17:302. doi: 10.1186/s12864-016-2602-9 (PMC4842269; doi:10.1186/s12864-016-2602-9)
Supplement: Additional file 4: — Supplementary methods. (PDF 1355 kb) [file 12864_2016_2602_MOESM4_ESM.pdf]

Additional File 4. Supplementary Methods.

Content | Page

De novo prediction of promoters in *B. japonicum* USDA 110 ……………………………………… 2

Overview …………………………………………………………………………………………………… 2

Step 1: Finding abundant patterns……………………………………………………………….. 2

Scoring scheme……………………………………………………………………………………. 2

Selection of α………………………………………………………………………………………. 3

Scoring of patterns and normalising for the GC-content of a pattern……. 4

Selecting over-represented patterns…………………………………………………… 6

Step 2: Clustering of the upstream regions using PCA………………………………………… 6

Score of a pattern in upstream regions………………………………………………… 8

Identification of top-scoring upstream regions……………………………………. 8

Principal component analysis to find different classes of upstream regions 8

Iterative algorithm to build a PWM………………………………………………………. 9

Calculating statistical significance………………………………………………………. 9

Step 3: Locating promoter sequences upstream of TSSs…………………………………….. 10

Calculating statistical significance………………………………………………………. 11

Re-annotation of the *Bradyrhizobium japonicum* USDA 110 genome……………………………. 12

Estimation of 5'- and 3'-UTR lengths………………………………………………………………… **15**

TSS distribution in intergenic regions ………………………………………………………………… 18

iTSS distribution in genes ……………………………………………………………………………… 19

Experimental procedures…………………………………………………………………………………… 20

Cloning procedures…………………………………………………………………………………………….. 20

RT-PCR ……. 21

qRT-PCR ………. 21

References……. 22

De novo prediction of promoters in *B. japonicum* USDA 110

Overview

The motif discovery was performed in three steps. At the first step, we aimed to find *patterns* (pairs of 6-mers separated by a spacer) over-represented in a given sample of sequences. Patterns were scored using a flexible scheme that allows for mismatches in sequence and deviations in position of both 6-mers. We selected 6-mers that occur together more frequently than expected given their individual frequencies by scanning all possible 6-mers at all positions. As the average GC-content of the genome is 0.64, GC rich 6-mers would occur more frequently by chance. To account for that, we normalized frequencies of patterns by their GC-content.

At the second step, *motifs*, that is, distinct clusters of overrepresented patterns, were identified with PCA; for each cluster, we then constructed a PWM representation (i.e. logo) for each motif. Finally, at the third step, we identify the highest scoring patterns for each motif in each TSS upstream region, and use these scores to select the relevant motifs.

Step 1: Finding abundant patterns

Scoring scheme

First, for each position in the 60-nt upstream region (total 55 positions for a 6-mer), we identified 30 most frequently occurring 6-mers using the following scoring scheme. The score of a given 6-mer at a given position is defined as $\alpha^{\mu+|\phi|}$, where μ and φ are the number of mismatches in the current sequence, and the offset in base pairs, respectively, and α is a parameter between 0 and 1 regulating the relative contribution of perfect sites and sites with mismatches; the choice choice of α is explained below. When scoring a single 6-mer, we considered only matches with $\mu + |\varphi| \leq 3$, i.e. such that the sum of the number of mismatches and the absolute value of the offset does not exceed 3. Since a 6-mer may have several ways to match at a given position in a given sequence, the maximum score of each 6-mer in each sequence was considered (see paragraph below).

An example of multiple successful matches would be an n-mer ATATAT in a sequence TATATATAGATA. When calculating the score of this 6-mer in this sequence at position 3, the sequence with no offset (TATATA) will have 6 mismatches. However, the best matches will be those with an offset -1 and 1: they would have 0 and 1 mismatches respectively, and would be assigned scores of α and α^2, respectively; the score of α will be recorded as the best score. Similarly, when calculating the score of the same 6-mer at position 4, we can find a match with offset 0 and 1 mismatch (score of α), or with offset -2 and 0 mismatches (score of α^2), or with offset +2 and 1 mismatch (score of α^3); the score of α would be selected as the highest one.

When scoring patterns (pairs of 6-mers), it is important to account for the fact that bacterial promoters have a preferred distance between the two boxes. Hence, the score is defined as $\alpha^{\mu+\nu+|\varepsilon|+\min(|\varphi|,|\psi|)}$, where μ and ν are the numbers of mismatches for the first (left) and second (right) 6-mer, respectively, ε is the extension of the pattern (change in the spacer length), and φ and ψ are the offsets of the left and right 6-mers, respectively (see the Fig. I below). The extension is

introduced in order to distinguish a shift of a pattern with intact distance between 6-mers, and a shift with a change in the spacer length (extension or contraction of the pattern).

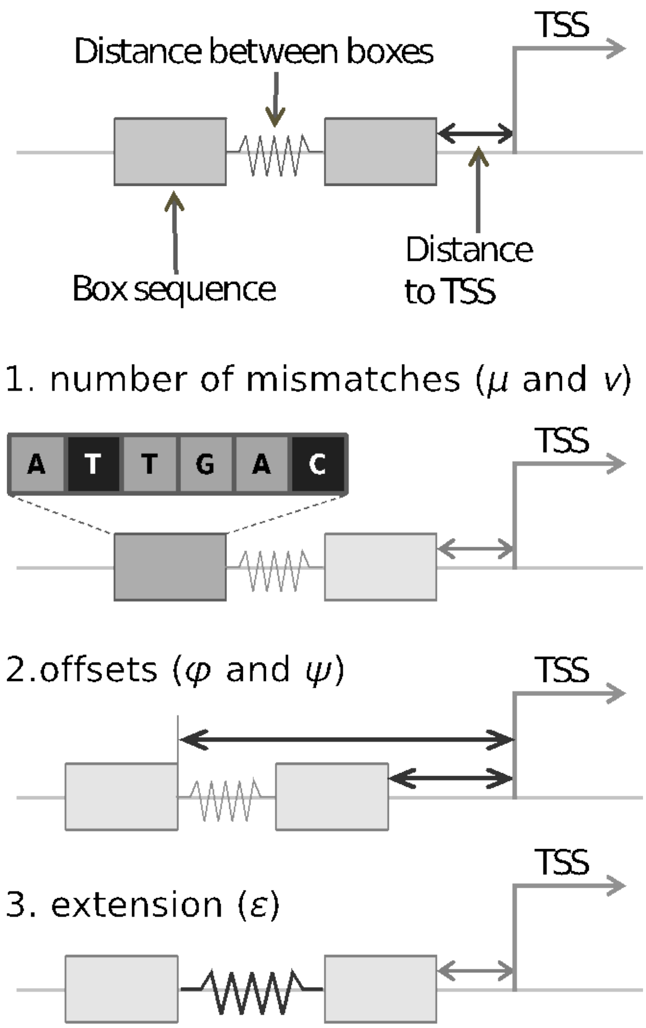

Figure I. Promoter scoring scheme. The pattern was aimed to find a best match in an upstream region. To accommodate the flexibility of the promoter structure, we allowed for deviations of three types, each of them equally penalized: (1) mismatches (μ and ν); (2) offset (φ and ψ); (3) extension

(ε).

A pattern is assigned a non-zero score if the number of mismatches of each 6-mer does not exceed 3 (to avoid cases with severe imbalance in the number of matches in the 6-mers); the offset and extension of the pattern are capped at 3; and the sum $\mu + \nu + |\varepsilon| + |min(\varphi, \psi)|$ does not exceed 5. We note that the number of mismatches was capped at 3 because, at GC-content of 0.64, the probability of two random 6-mers matching with 4 mismatches is equal to 30%.

Selection of α

The total score of a 6-mer (the sum of scores in all upstream regions) aims to reflect the number of its (imperfect) occurrences. While a perfect match in any case counts as a single occurrence (α^0=1), any match with a mismatch/offset/extension counts with a fractional score; the coefficient α, 0<α<1 controls how much do imperfect matches contribute to the total score. This coefficient is adjustable;

if it is close to 1, there are only weak penalties for offsets and mismatches, whereas if α is close to zero, non-perfect matches are assigned very low scores (<<1), and the total score basically counts the number of perfect matches that are rare and hence do not provide an adequate statistics.

Increasing α would increase the total score of a given 6-mer or a pattern. To select the optimal value of α, we increase it until the scores of patterns reach around 20 for the smallest dataset used. This means that a perfect match has a weight of less than 5% of the total score, and thus we can believe our scores with uncertainty of the order of 5%. To verify that this error is sufficiently small, we have calculated the difference between the top score and 500th score in our list. The difference was much more than the relative weight of the perfect match (5%). This ensures that our selection of best-matching patterns is not dominated solely by perfect matches, and incorporates sufficient information from imperfect matches with mismatches and offsets.

Scoring of patterns and normalizing for the GC-content of a pattern

The number of possible patterns is more than a billion. The number of possible mismatches and offsets is also large (of the order of 1000 different combinations of mismatches and extensions). Hence, scoring each pattern in each upstream region is computationally unfeasible. Therefore, we consider only 30 best 6-mers at each position, which gives 900 patterns (30x30 pairwise combinations) formed by these 6-mers *at each pair of positions*. At that, the total number of patterns we analyzed is 1,102,500=900·1225, where 1225 is the number of pairs of 6-mer positions, overlapping by no more than 1 bp, in a 60-bp upstream sequence.

The next step is selecting pairs of 6-mers that co-occur more frequently than by chance. We have started with a scatter plot whose X-axis corresponds to the product of scores of two 6-mers, and the Y-axis corresponds to the score of the pattern formed by these 6-mers. We have observed a strong correlation between the two: if both 6-mers are frequent, then they are more likely to occur as a pair. In addition, we observe that the score of a 6-mer or a pattern strongly depends on its GC-content, see the top panel in Fig. II below, where GC-rich (red) patterns have higher scores than GC-poor (blue) ones.

To account for that, we developed a normalization technique. We note that while very GC-rich 6-mers are abundant, they do not represent any known motifs. Therefore, 6-mers with 0 or 1 A/T were excluded. The remaining 6-mers were split in 4=6–2 groups by the number of G/C. The average abundance of 6-mers from each group was calculated. A vector of average abundances was then mean-normalized, and the abundance of each 6-mer was divided by the normalized average abundance in a group to which this 6-mer. These 6-mer normalized abundances were used to select highest scoring 6-mers at each position. Mean-normalization of the correction vector does not affect selection of the best 6-mers; however, it ensures that the scores after correction retain their meaning of an approximate number of perfect matches, and thus can be interpreted when troubleshooting. The score of a pattern is similarly divided by the product of correction factors for each of the two 6-mers forming this pattern.

The bottom plot in Fig. II (see below) shows scores of patterns vs. products of 6-mer scores after correction for GC. Each pattern is colored by its cumulative GC content, similarly to the plot on the left. However, here the colors are no longer separated, indicating that our normalization have successfully removed biases associated with GC-content.

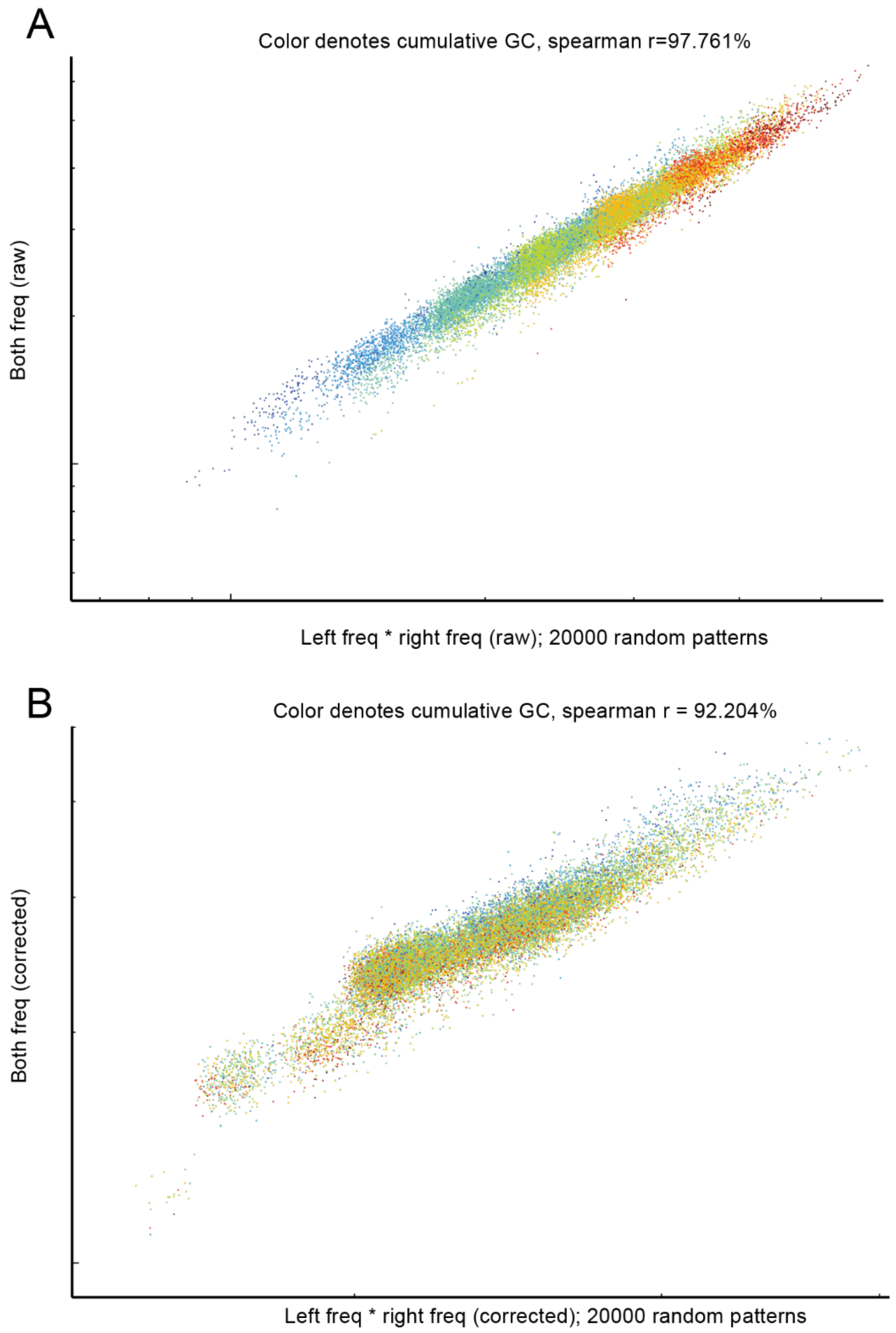

Figure II. Correction for the GC-content bias. (A) The scatter plot shows how the product of the raw scores for the two 6-mers forming a pattern (x-axis) is related to the raw score of the pattern, i.e. two 6-mers occurring together (y axis). Scores shown are for a full set of TSSs. Both axes are log-scale. Because the number of analyzed pattern is very high (1,102,500), for this plot we randomly selected 20,000 patterns. Color of each point shows the sum of GC-contents of the two 6-mers making the pattern; blue means low GC-content, red, high GC-content. Note that both pattern score and the product of two sub-scores strongly depend on the GC-content, which can be seen as separation of colors along the cloud of points. (B) A similar scatter plot, but with scores on both axes corrected for the GC-content bias. Note that there is no separation of colors anymore, proving that the correction effectively removed biases associated with GC-content.

Selecting over-represented patterns

If the relationship between the score of a pattern and the product of individual 6-mer scores were purely linear, it would be reasonable to consider the ratio

$$\frac{pattern\ score}{6-mer\ score_{left} * 6-mer\ score_{right}}$$

and select patterns with the highest value of this ratio. Yet, if done so (see the top panel in Fig. III below), the non-linearity often occurring in the scatter plot (colored bulge on the scatterplot) would capture top 3000 patterns. This non-linearity was most abundant in the “equally” class. These patterns occur by chance more than expected, yet they have a very low overall frequency. They likely emerge due to a slightly non-linear relationship between the pattern score and the product of 6-mer scores, or are due to the sampling noise. To compensate for this, we use a slightly modified score, which gives more weight to patterns that occur frequently:

$$score = \frac{pattern\ score^{\sqrt{3}}}{6-mer\ score_{left} * 6-mer\ score_{right}}$$

This scoring is logical when considered in the log-log space. Note that a linear relation y=a·x+b is generally not a straight line in the log space; instead, straight lines have the form $x^a \cdot y^b = const$ (equivalent to $\log(x) + \log(y) = const$). The line $y = a \cdot x$ has a slope of 45° in the log-log plot. The scoring schema we use corresponds to shifting the cut-off direction by 15°, from 45° to 30°, as $\sqrt{3} = \frac{\cos(30°)}{\sin(30°)}$

Step 2: Clustering of the upstream regions using PCA

After selecting top 500 patterns, we aimed to see whether patterns belonging to different clusters describe different types of promoters. Comparing patterns directly may be complicated because 6-mers may be shifted relative to each other, or redundant (e.g. if a 6-mer describes a 4-mer motif, there will be 16 possible 6-mers contributing to the same signal). Instead, we compare patterns using their scores in the list of upstream regions.

First, we drop all positional information from patterns, and retain only unique pairs of 6-mers. The rationale for this is that the downstream algorithm will be distinguishing between different motifs. However, different locations of binding sites within a selected 7-bp window could mislead the algorithm into focusing on different locations, rather than different binding preferences. Specifically, two patterns composed of exactly the same 6-mers, but offset by one or two nucleotides, will give substantially different scores to the sequences with exactly the same binding sequence at slightly different locations. The number of unique pairs of 6-mers without positional information was not much less than the 500 initially selected patterns, usually about 300.

Additionally, we focused only on patterns that map to a certain location of upstream regions. Specifically, for all upstream classes but “Nod only” we found that all of the top 500 patterns were located near the canonical sigma-70 position, while for the “Nod only” class, 499 out of the top 500 patterns were located near the sigma-54 position. Consequently, for “Nod only” class we focused on

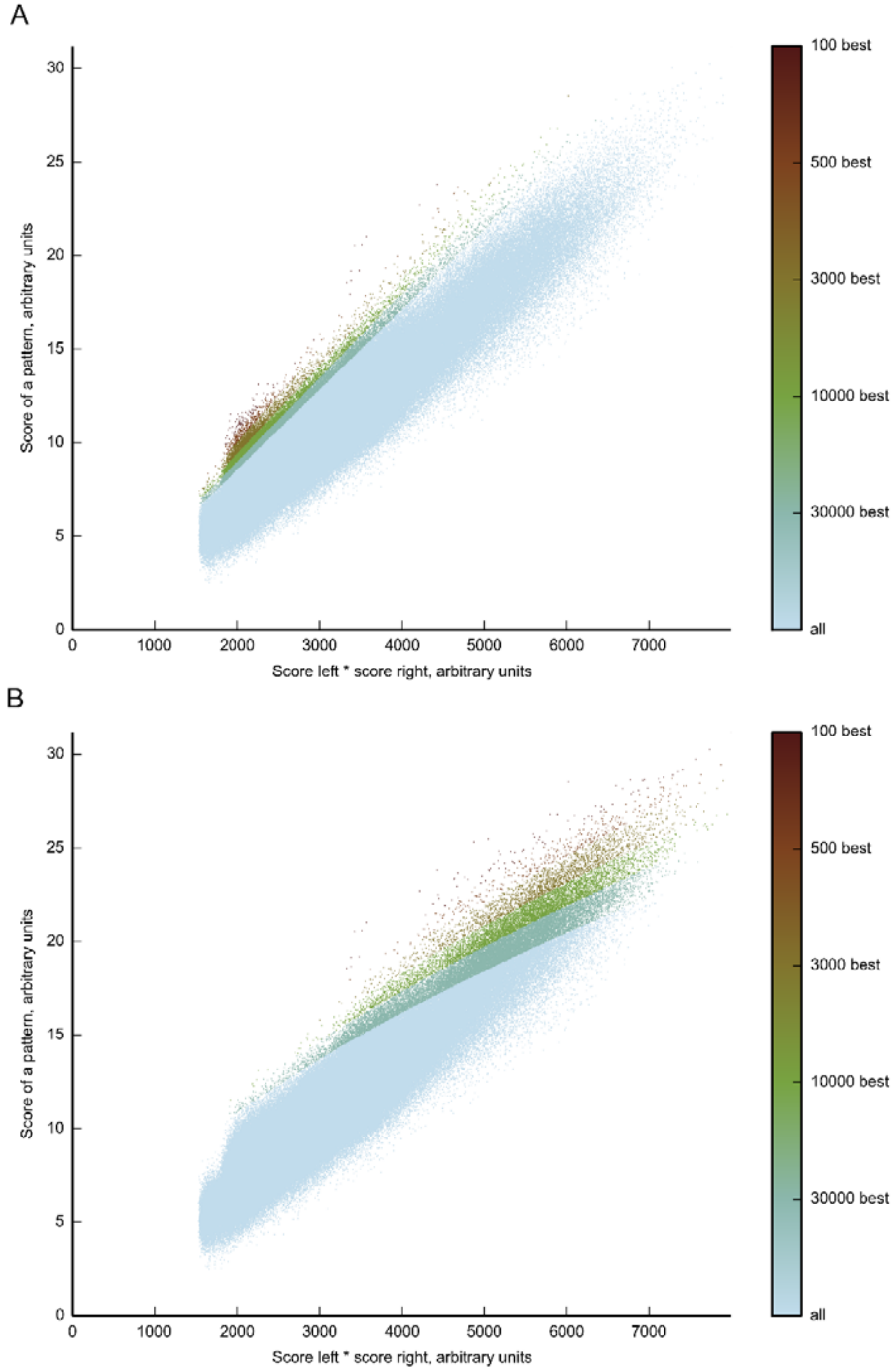

Figure III. Criteria for selection of over-represented patterns. A scatter plot similar to Fig. II (see above), but now colored to indicate top patterns selected with different selection criteria. Both plots are for the “Equal” class of upstream regions, because the bulge has been very prominent in this class. In other classes the bulge was often present, and partly selected using the naïve selection criteria. (A) The naïve way of selecting over-represented patterns, which score more than the product of the two sub-scores for the two 6-mers making the patterns. In this case, top-scoring patterns are diluted by a sub-population of low-scoring patterns in the bulge. (B) An improved selection where slightly more weight was given to patterns occurring more often. Now patterns from the bulge are not included in the selected set.

patterns within 3 bp from the sigma-54 position, (–28±3, –17±3), and for others, within 3 bp from the sigma-70 position, (–36±3, –13±3). These positions were inferred from the distribution of locations of the left and right 6-mers, and highlight the peak of this distribution. We note that the positions presented here indicate the start of a 6-mer, and not the start of the known promoter sequence. Hense, especially for a shorter right box, the numbers above are slightly less than the canonical positions of the promoter sequences.

Score of a pattern in upstream regions

We score each pattern in the ±3bp windows defined above. At that, we do not penalize offsets (unlike above) and allow for up to three mismatches for each box, with the maximum allowed number of mismatches set to 5.

The score of a pattern in a given upstream region is thus defined as

$$5 - (total\ number\ of\ mismatches);$$

a higher score now indicates a better match. Patterns that do not match the criteria (have more than 5 mismatches, or more than 3 mismatches per 6-mer), are assigned a zero score. Perfect match has a score of 5. The resulting matrix has dimensions

$$(Number\ of\ unique\ patterns\ < about\ 300 >) \times (Number\ of\ upstream\ regions).$$

Identification of top-scoring upstream regions

As matrix of scores build at the previous step is discrete, selecting the top upstream regions by the best score may be ambiguous. To avoid this problem, we use the k-power average, $\sqrt[k]{(x_N^1 + \cdots + x_N^k)}$ that has the limit of max(x_1, ..., x_N) as k goes to infinity. For a finite but large k, the k-power average is dominated by the maximum score, but provides a bonus for having scores close to the maximum. We use the 10-power average because it produces a relatively smooth histogram of scores. For the 20-power average, the distribution of average scores has peaked around 3,4,5, indicating that it is already very close to simply taking the maximum.

Principal component analysis to find different classes of upstream regions

Visual inspection of the matrix of scores indicates that about a third of upstream regions have a distinct site of a given sigma-factor. For the downstream analysis we use the top 33% upstream regions, but not less than 200.

We apply Principal Component Analysis (PCA) in the space of upstream regions to find directions along which the matrix of scores changes most distinctly. We assume that the most distinct upstream regions along each principal component have a different motif. For each principal component we select top and bottom 200 (or a half if the number of upstream regions is less than 350) upstream regions (those having a highly positive and a highly negative coordinate along this axis) and use them to build a positional weight matrix (PWM). To ensure the convergence of the PWM-building algorithm (see below), a seed pair of 6-mers is needed; to identify those, the matrix is projected onto a given principal component, that is, the dot product of the matrix with the principal component is taken. It yields a vector whose length equals the number of patterns. The minimum and maximum of this vector are the seeds for the two groups of upstream regions, respectively.

Iterative algorithm to build a PWM

For each group of 200 sequences selected using PCA, we then build a PWM consisting of two 8-mer boxes. The length 8 is used instead of 6 to capture longer motifs, e.g. TGNTATAA for leaderless transcripts. In addition to probabilities of each nucleotide at each position of the two boxes, the PWMs also contain probabilities of extensions and locations of the right motif. This allows us to capture preferences in the location of the promoter relative to TSS.

We start with a seed PWM, generated as follows. All locations and extensions are assigned equal probabilities. The first and last nucleotides are assigned probabilities of 0.25. The middle 6 nucleotides of each 8-mer are seeded with the respective 6-mer from the best-matching pattern. Nucleotides which match the 6-mer are assigned probability of 0.7, and the remaining nucleotides are assigned probability of 0.1. (Note that changing the value of 0.7 will not change the results, because it will not change the ranking of upstream regions or positions of the best match during the first pass. Yet it is important that we start with a seed motif, rather than with a random PWM, and it is important to have non-zero values in the PWM; zero values would make it impossible to distinguish between 1 and 2 mismatches.).

The seed PWM is then scored in all selected (usually 200) upstream regions at all possible locations and extensions. The maximum score for each upstream region and the respective site are recorded. If (rarely) two sites are assigned the same score, a random one is selected. All 200 upstream regions are then ranked by their score and weighted proportionally by their position so that the top ranking upstream region is assigned weight 1, and the bottom ranking one, 0. Frequencies of each nucleotide at each position are calculated using the recorded sites and taking into account these weights. This calculation also uses a pseudo-count of 1 and normalization by the genome-wide GC-content of 0.64. Similarly, frequencies of positions and extensions are calculated with the same weighting scheme with a pseudo-count of 1. A PWM is then updated using the calculated frequencies, and this procedure is repeated 40 times, sufficient for convergence.

The resulting PWM was scored in all (~15000) upstream regions. A significant fraction of regions used for calculating a PWM was recovered in the top 200 hits; for the “Nod only” of “full” groups this fraction was around 0.3-0.6.

Calculating statistical significance

To calculate statistical significance of the resulting score, we scored the PWM in control sequences. To create a control sequence, we shuffled nucleotides at each position of the upstream region in a full list of regions; specifically, we selected the first nucleotide randomly from first nucleotides of all upstream regions; second nucleotide from all second nucleotides, etc. We created 40000 control sequences. For each real sequence, we then defined the p-value as the probability of obtaining the score of the real sequence in the control sample; specifically, we set

$$p\_value = \frac{1 - rank(score, controlScores)}{40000}.$$

Step 3: Locating promoter sequences upstream of TSSs

We then aimed at the next goal, finding promoter sequences in the considered upstream regions. PCA is best suited to cluster promoter sequences of a certain types of motifs. However, to locate promoter in a sequence, we used a more direct approach, which integrates information from all top overrepresented patterns obtained at step 1, and thus encompasses all motif types.

In brief, for each pattern, we found upstream regions in which the pattern scored well. However, when the pattern scores in a given location, not all 6 positions of each box of the pattern are equally important. For example, a pattern TTGACA, which scored for the left box of the sigma-70 factor, would more often have mismatches in the ACA part, and would almost always match TTG perfectly (given that it matches well). To find out which part of the 6-mer is actually important for binding, we evaluated Information Content (IC) of each nucleotide of each pattern. We then recorded which promoters have contributed to the high-IC positions of this pattern. We repeat this for all 300 best patterns; after that, for each position of each upstream region, many patterns could have contributed with different IC. We took top 10 patterns contributing to each position in each upstream region (if there were at least 10), and evaluated the average IC at that position. If it was above a threshold, we assumed that this position in this upstream region belongs to a promoter.

Below, we outline an exact algorithm used to highlight the promoter sequences.

1. Best 300 patterns (pairs of 6-mers with positional info) were selected for each class of upstream regions (Nod only, Equal, etc). Those were then scored in all upstream regions (i.e. in the "full" dataset). For each pattern in each upstream region, the best match was selected; if several best matches occurred (which happened rarely), a random one was selected. In addition, location of the left and right boxes for a selected match were recorded.

The next two steps worked with a matrix (15000 x 300) of best scores of 300 patterns in all upstream regions, and the corresponding matrices of positions of the best matches of the left and the right box.

steps 2-5 were performed for each pattern

2. For each pattern, we selected upstream regions, which were assigned a score at least 4. The score was defined the same way as during pattern selection (# mismatches + offset + extension). We then aimed to determine which bases of the two 6-mers in the pattern are contributing to the structure of the promoter. We did this by constructing a PWM (see next step). A PWM constructed from matches of patterns in upstream regions would contain information about which positions in a 6-mer usually match perfectly, and which are frequently substituted.

3. For each pattern, we build a PWM using selected upstream regions (score at least 4) from a given dataset (e.g. “Nod only”, if best patterns from “Nod only” were used). PWM was build using a single-pass procedure, not to be confused with the iterative algorithm described above. In particular, in selected upstream regions, we counted frequencies of nucleotides in 8-mers centered at left and right boxes (6-mer boxes were extended on both sides by one nucleotide). For control, we counted frequencies of nucleotides in all (full) sequences at shuffled positions. Shuffling was done as follows: for each upstream region in the control set, we selected a random position of the left and right boxes from those used to construct the actual PWM. Left and right positions were selected independently.

The goal of this was to capture nucleotide and dinucleotide composition of the regions where a given pattern scores, but not to capture any correlations between the left and right box in the control sequences. We then divided the real frequencies by the control frequencies. The resulting relative frequencies were then normalized so that they summed to one at each position.

4. For each PWM (i.e. each pattern), we then calculated Information Content (IC) at each position of each box. IC was determined as $\sum_i P_i * ln(P_i / 0.25)$. Since P_i are normalized to sum to one, this would be a number between 0 and $ln(4)$.

5. Now we focus again on all sequences. For those sequences which were selected by a given pattern (with score less than 4), per-nucleotide IC was recorded for nucleotides covered by left and right boxes.

6. Now for each sequence we have some number of ICs recorded at each position (each IC record at each position may come from some set of patterns, but of course not all patterns scored in all sequences at all positions). For those nucleotides that had at least 10 patterns contributing to them, we then calculated the average of top 10 ICs values. We decided to use the average over top 10 patterns to focus on promoters that scored high in at least some patterns. Taking an average over all patterns would select most “mediocre” promoters that scored well in all patterns they scored at. Averaging over best 10 was selected to avoid drawing conclusions from a single 6-mer, as scoring of a single 6-mer may be a coincidence.

7. We now set three different cutoffs on ICs: 0.3, 0.45, 0.6. For each pattern, positions where the average of top 10 ICs was more than the cutoff, were labeled with capital letters.

8. In the resulting tables, upstream regions were ordered by the score, which was calculated as follows: $6 - \sqrt[10]{((\sum_i (6 - score_i)^{10})/10}$, and the mean is taken over best 300 patterns. This is the 10-power average of the score, and is equivalent to taking the *smart minimum* score. If two upstream regions have an equally good minimum score, then the one that has more patterns with second best score would be favored. The same type of averaging was used previously. We note that this ordering is independent from the information algorithm used to identify promoters, and thus upstream regions with identified promoters may have lower scores that upstream regions with no promoters in the table. However, the ordering separated upstream regions with identified promoters, and upstream regions without, reasonably well.

Calculating statistical significance

Here, we created shuffled upstream sequences as defined above. We then used the scoring procedure from section 8 above to calculate the statistical significance. We performed this scoring procedure in given sequences and in control sequences, and defined the p-value as the probability to obtain the given score in control sequences, i.e. $p = 1 - \frac{rank(score,controlScores)}{} 40000$. We used the cutoff of p=0.1 for the full set of sequences, and p=0.05 for different subclasses.

Re-annotation of the *Bradyrhizobium japonicum* USDA 110 genome

Existing methods for bacterial gene prediction perform relatively well, but errors are not uncommon (Ederveen et al., 2013). Annotation of bacterial genomes is at least partially automatic and often introduces long open reading frames, especially in bacteria with high GC-content (Hyatt et al., 2010). As *B. japonicum* USDA 110 is GC-rich (64 % GC-content) and longer ORFs are a likely source of numerous false iTSSs, which are in reality gTSSs, we decided to re-annotate the genome.

An updated and extended annotation of the *B. japonicum* USDA 110 genome was generated in July 2013 by submitting its genomic sequence to the Ergatis pipeline of Integrated Services of Genomics Analysis (ISGA) (Hemmerich et al., 2010), and to RAST, another automated genome annotation engine (Aziz et al., 2008). While the resulting annotations were largely consistent (see Table I below), when compared to RAST, ISGA yielded more genes and shorter reading frames (see Fig. IV below). Furthermore, less genes of the original RefSeq annotation (Kaneko et al., 2002) were questioned by ISGA than by RAST (see Tables I and II below). ISGA is also highly assessed in comparative studies of annotation engines (Ederveen et al., 2013). For these reasons we used the ISGA annotation as the base for TSS-to-gene mapping. We provide annotation files in gbk and gff file format (Additional files 5 and 6).

We preserved the original gene identifiers (locus tags) of the RefSeq annotation by Kaneko et al. (2002). When ISGA predicted a shorter or longer form of the same protein, "_sh" and "_ln" were added as a suffix of the locus tag (e.g. blr1613_sh). For genes predicted only by ISGA, we used locus tag notation as in Kaneko et al. (2002), numbered them independently (e.g. bll0001_ISGA). In the ISGA annotation 523 of the original RefSeq genes were assigned as questionable, 3050 changed only their start-codon position, typically resulting in a shorter ORF (see Fig. V below), 4798 genes preserved their predicted boundaries and 1351 new genes were predicted (see Table I below).

We enhanced the gene annotation with proteomic evidence. We added note: "protein supported by proteomic data" to gene feature in the gbk and gff files (Additional files 5 and 6). When peptides distinguishing shorter and longer form of the protein were identified, we kept the version with proteomic evidence (see Table 1 in the main text). For seven proteins, both peptides for longer and shorter form (RefSeq and ISGA annotation) were present, so both protein forms were retained in the annotation. Additionally, 39 proteins present in RefSeq annotation only, were restored (see section "Protein translation evidence for TSSs data" and Table 1 in the main text). Thus, in addition to 9,199 genes annotated by ISGA (see Table II below), 46 more genes were included in the provided annotation gff and gbk files (Additional files 5 and 6), resulting in 9,245 annotated genes. Importantly, our new annotation increases the fraction of transcription start sites, which can be attributed to protein-coding genes (see Fig. V below).

Our annotation also includes Rho-independent terminators predicted using the tools ARNold, WebGesterGB and TransTermHP (Naville et al., 2011; Mitra et al., 2011; Kingsford et al., 2007).

Having terminators in annotation aids to assess likely sizes of transcripts and select appropriate primers for the validation procedure.

We adapted operons as predicted in the ProOpDB database (Taboada et al., 2012) to the new gene annotation. The adapted operons were used for expression statistics (see "TSS categorization" in the main text and Additional file 1: Table S2). Operons were adapted as follows: genes with changed start were assigned to the same operon; genes with new annotation yielded a new independent operon. Each operon was assigned an operon ID "Bja_Operon_XX", where XX is the index number of the operon, and was added to the annotation file as well.

Newly identified elements, TSSs and promoters, were also included into the annotation file. Each TSS was assigned a locus tag: Bja_TSS_XXXX, where XXXX is the number, in order of appearance in the genome. Each promoter has a note about the promoter type (e.g. RpoD, RpoN) and the respective TSS.

The annotation was combined using Biopython 1.65 (Cock et al., 2009), and converted from the gbk to the gff format by Geneious version 8.1 (http://www.geneious.com, Kearse et al., 2012).

	ISGA vs. RefSeq	RAST vs RefSeq	RAST vs. ISGA
matching CDSs	4,751	4,669	7,690
matching genes	4,798		
re-annotated start	3,050	2,941	898
New genes or CDS	1,351	1,105	556
questioned	523	707	127

Table I Comparison of different annotations. Genes include CDS, rRNA and tRNA genes. RAST predicts only CDS (ORFs) only.

	RefSeq	ISGA	RAST
genes	8,373	9,199	
CDS	8,317	9,146	8,715

Table II Number of genes and CDS predicted in each annotation. RAST predicts CDS (ORFs) only. Genes include CDS, rRNA and tRNA genes.

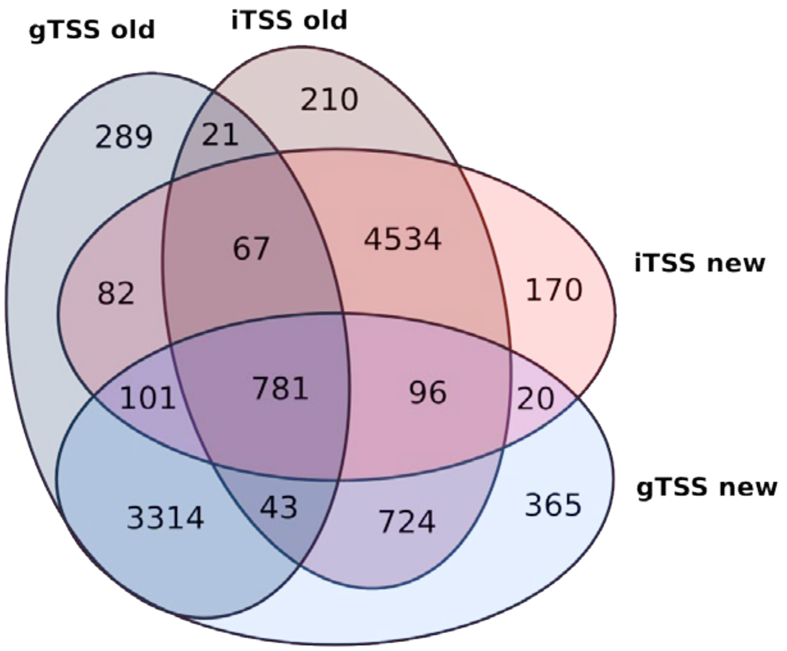

Figure IV. Venn diagram of TSSs, mapping immediately upstream of a gene (gTSS) and inside ORF (iTSS). New - mapping with respect to ISGA annotation, old - with respect to RefSeq annotation.

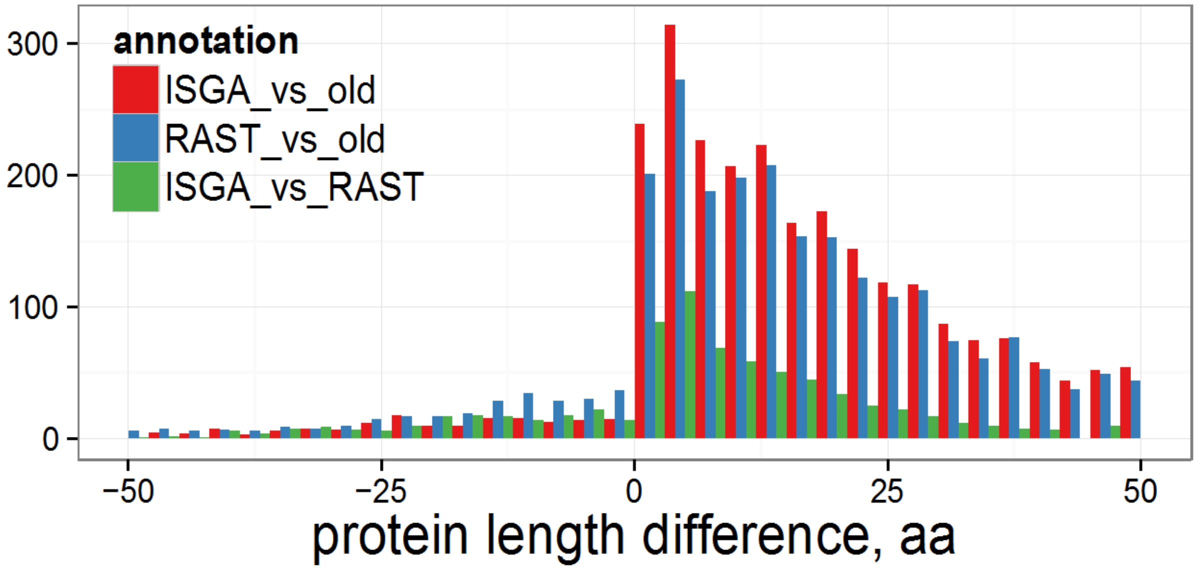

Figure V Numbers of genes with changed ORF length. ISGA or RAST re-annotation of *B. japonicum* USDA 110 genome is compared to the RefSeq annotation by Kaneko et al., 2002 (old).

Estimation of 5’- and 3’-UTR lengths

To estimate 5’- and 3’-UTR parameters, only transcriptional start sites (TSSs) mapped to intergenic regions (IGRs, regions between annotated genes) were considered. This assumes that TSSes that both map inside a gene and at the same time are the start site of a mRNA, sRNA or an anti-sense RNA are rare.

First, we analyzed the distribution of predicted 5’-UTR (leader) lengths, i.e. the distance from a TSS to the start of the downstream gene or ORF (see Fig. VI below). When multiple TSSs were present, we used the distal TSS mapped upstream of the annotated gene in the sense orientation. We found that 5’-UTRs in both divergons and synvergons (for the definition of divergon and synvergon see Fig. VIII below; Tsoy et al., 2012) are typically 20-40 nt long and rarely exceed 200 nt (see Fig. VI below showing the data used for determination of the length of 5’-UTR for categorization purposes). Therefore, we categorized TSSs located within 200 nt to the start codon as a gene TSS (gTSS) or as a TSS antisense to the 5’-leader (aTSS_5) for the sense and anti-sense orientations, respectively (see Fig. 1B in the main text).

The definition of a cutoff for 3’-UTRs is necessary for the categorization of TSSs that are antisense to 3’-UTRs (aTSS_3). However, the genome-wide analysis of 3’-UTRs is difficult, because the location of the transcription terminator site is usually unknown. As a proxy, we assumed that, as the antisense RNA should overlap with the respective mRNA, aTSS_3 should be often located at a short distance from the mRNA end and, by implication, from the stop codon. On the contrary, independent RNAs encoded on the antisense strand should reside at some distance from the mRNA and the stop-codon of its ORF. Indeed, the distribution of the distances from a stop codon to the nearest downstream TSS in the anti-sense direction showed a prominent peak at 20-30 nt (see Fig. VIIA below). Importantly, for synvergons (see Fig. VIII below), which belong to a polycistronic mRNA, aTSS_5 and aTSS_3 definition is simply invalid, because asRNA may regulate several genes of the same operon; this is why short 3’-UTR prevalence effect disappears, when 3’-UTR is calculated in the relative scale (compare Fig. VIIA and VIIB below for synvergons). Therefore the 3’-UTR threshold was set to 100 nt based on the distance distribution in convergons only. To solve the problem of aTSS_5 and aTSS_3 in synvergons (in 70 % cases those IGRs fall into predicted operons), we introduced a new category called aTSS_op for aTSS, mapping between genes of one operon; “op” stands for “operon”. This category is used in the table listing all mapped TSSs (see Supplementary File S1, Table “Mapped TSSs). In TSS category statistics they are counted as aTSS (see Fig. 1C in the main text).

We note that many TSSs can be assigned to several categories (the above-mentioned aTSS_5 and aTSS_3, as well as iTSS and gTSS, two gTSS of different genes if the genes are closely located). Accordingly, 15,923 TSSs were mapped, but 20,071 TSSs are listed in see Additional file 3: Table S3. The coordinates of TSSs assigned to several different types are identical.

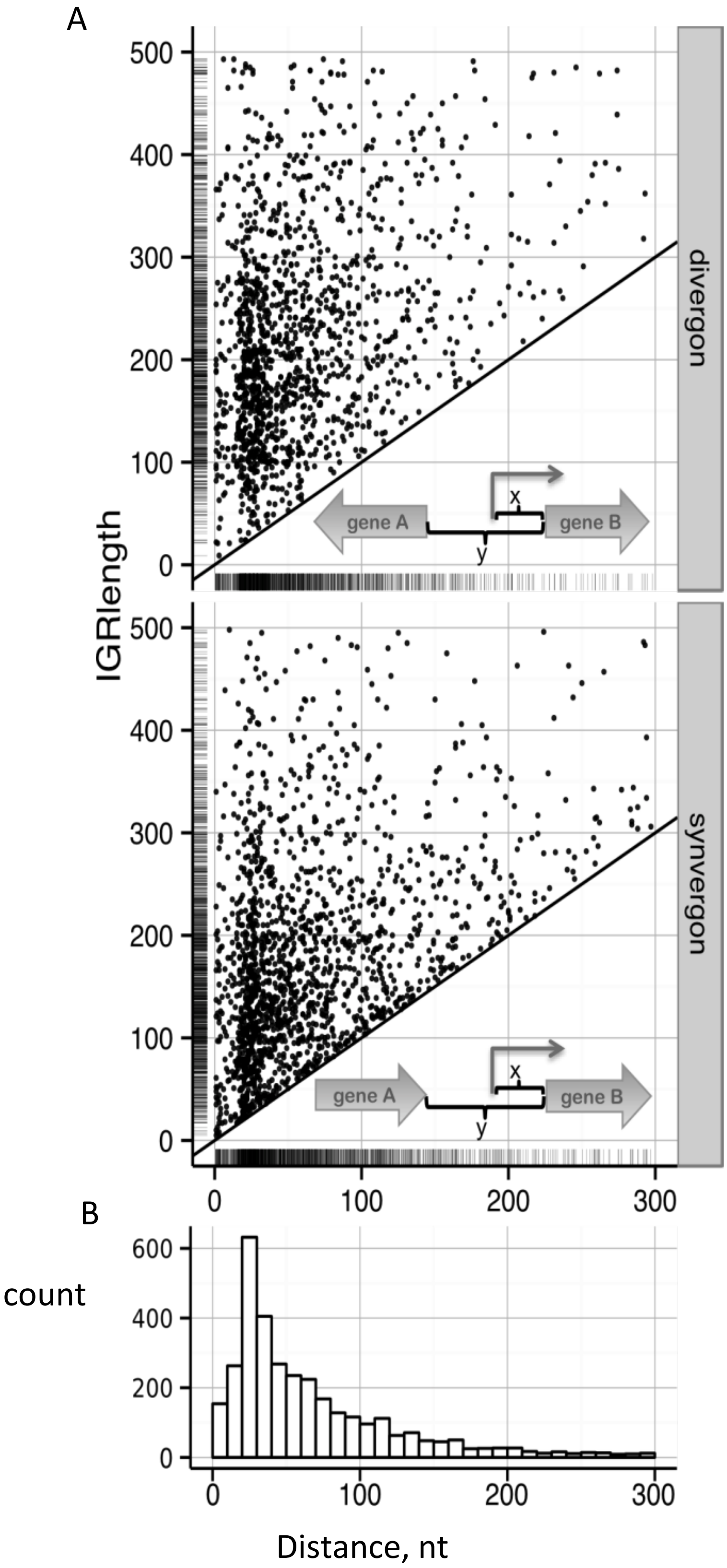

Figure VI. Genome-wide analysis of the distances from the distal TSS, mapped upstream of the annotated gene in the sense orientation, to the annotated start of the corresponding ORF in divergons and synvergons. These results were used for determination of the 5'-UTR length for categorization of TSSs. **A)** Distance between the distal TSS and a gene (x) was plotted against the IGR length (y). **B)** Graphical summary of the results shown in A).

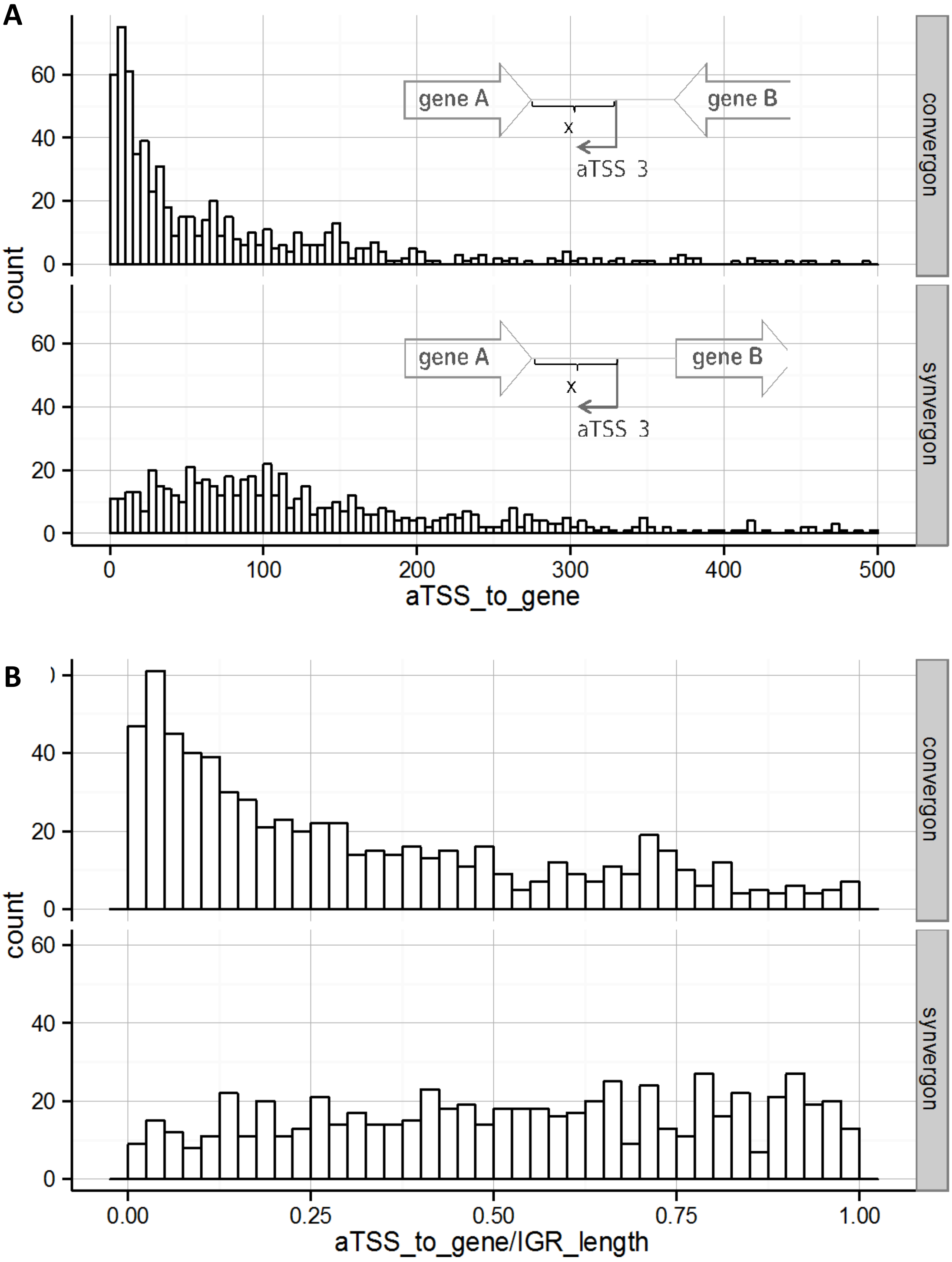

Figure VII. Distribution of distances from the end of a gene to potential aTSS_3. Here aTSS_3 is anti-sense oriented TSS, closest to the gene end; x is equivalent to aTSS_to_gene, measured in nt. These results were used for determination of the 3'-UTR length for categorization of TSSs. **A)** Absolute distances are skewed towards gene ends for convergons, and quite uniformly distributed for synvergons. **B)** Relative distances show, that, although for both types of IGRs distribution is skewed towards 0-150 nt range, for synvergons it is mostly due to short synvergon lengths. This suggests that the 3'-UTR threshold should be set based on distance distribution in convergons, and equal to 100 nt.

TSS distribution in intergenic regions

We analyzed the distribution of positively scored TSSs in intergenic regions (IGRs). Neighboring genes of *B. japonicum* USDA 110 usually have the same direction (64 % of the genes), and thus their corresponding IGRs are mostly synvergons. In agreement with the view that bacteria experience high evolutionary pressure to maintain compact genomes, we found that IGRs tend to be small (median/average 90/145.7 bp, see Fig. VIII below). Most IGRs in which we mapped TSSs are about 100-200 nt long (see Fig. VIII below), sufficient to accommodate *cis*-regulatory elements such as transcription factor binding sites and promoters.

We also analyzed the orientation of the mapped TSSs with respect to the nearest downstream gene and found that TSSs mostly map in the sense orientation. For example, although the number of convergons and divergons is essentially the same, 1501 and 1500 respectively (the difference is caused by the virtual linearization of the circular chromosome), the number of divergons with at least one TSS in it exceeds the number of convergons with TSSs (1071 and 533, respectively). Furthermore, 1,388 synvergons have TSSs only in the sense and 215 only in the antisense orientation, while 446 have TSSs in both orientations and 2,886 have no TSSs. This shows that *anti-sense* transcription in IGRs is much less abundant than ordinary mRNA transcription.

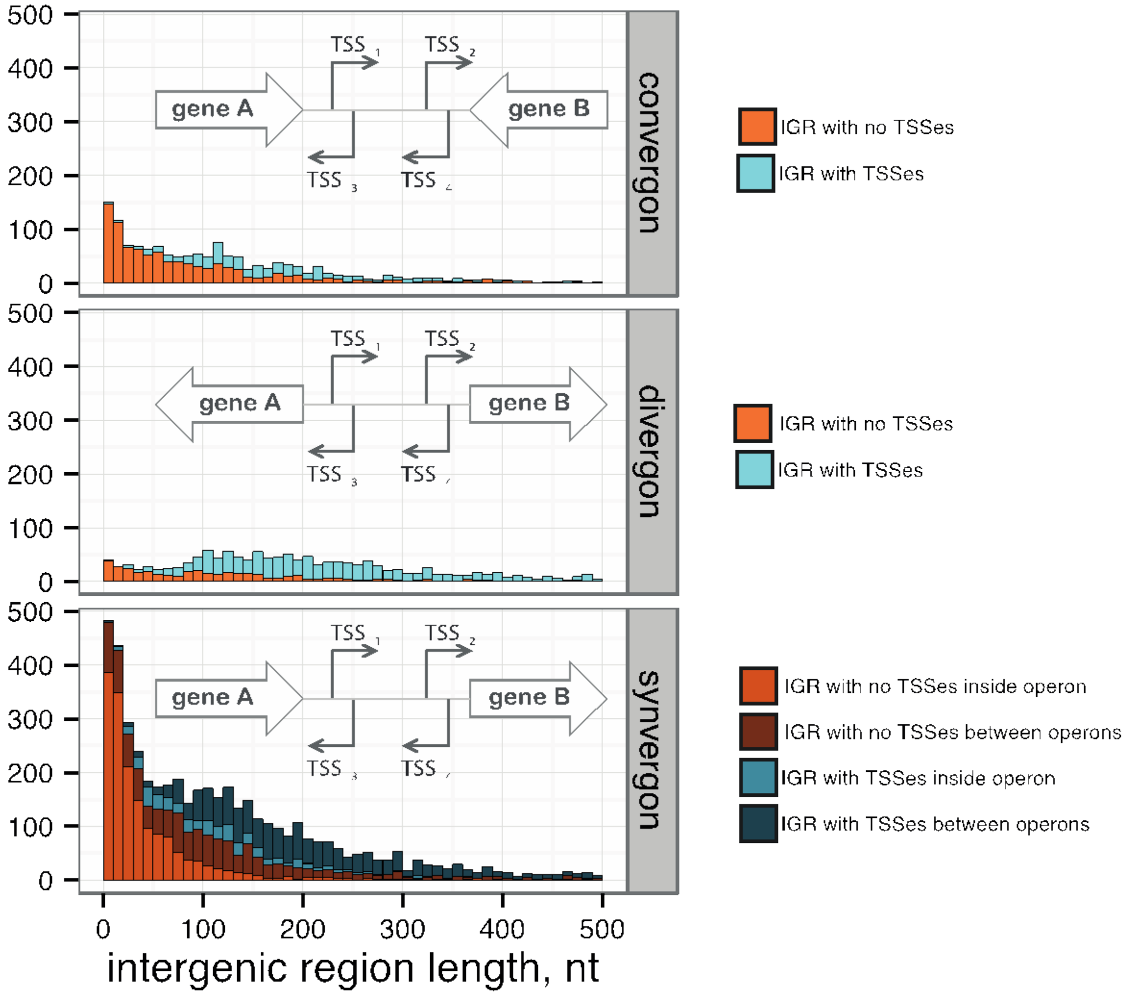

Figure VIII. Length distribution of intergenic region, conditioned on TSS mapping. Intergenic regions are divided in convergons, divergons and synvergons.

iTSS distribution in genes

iTSSs are the most abundant category of TSSs in this study and the question arises whether this is due to gene misannotation. As mentioned above (see Page 12, "Re-annotation of the *Bradyrhizobium japonicum* USDA 110 genome"), ISGA yielded more genes and shorter reading frames. This resulted in 75 more gTSSs and 41 fewer iTSSs.

Considering the possibility that still some iTSSs could be misannotated gTSSs located at the ends of genes, we analyzed the distribution of iTSSs in genes of the re-annotated genome. Fig. IXA below shows the relative distance of an iTSS from the start codon. It revealed clustering at starts and ends of genes. To exclude that this result is an artifact of short ORFs, the absolute distances of iTSSs from starts and ends of genes were also analyzed (Fig. IXB and IXC below), confirming clustering in the first 30 bp and the last 30 bp of genes. The clustering of iTSSs in the first 30 bp of genes suggests that even after re-annotation, some genes are shorter than annotated; clustering at the end of genes indicates that some iTSSs are probably TSSs of downstream genes.

However, Fig. IX also shows that the vast majority of iTSSs is distributed quite evenly in genes – most mapped iTSSs are located at different positions in genes and represent genuine iTSS candidates.

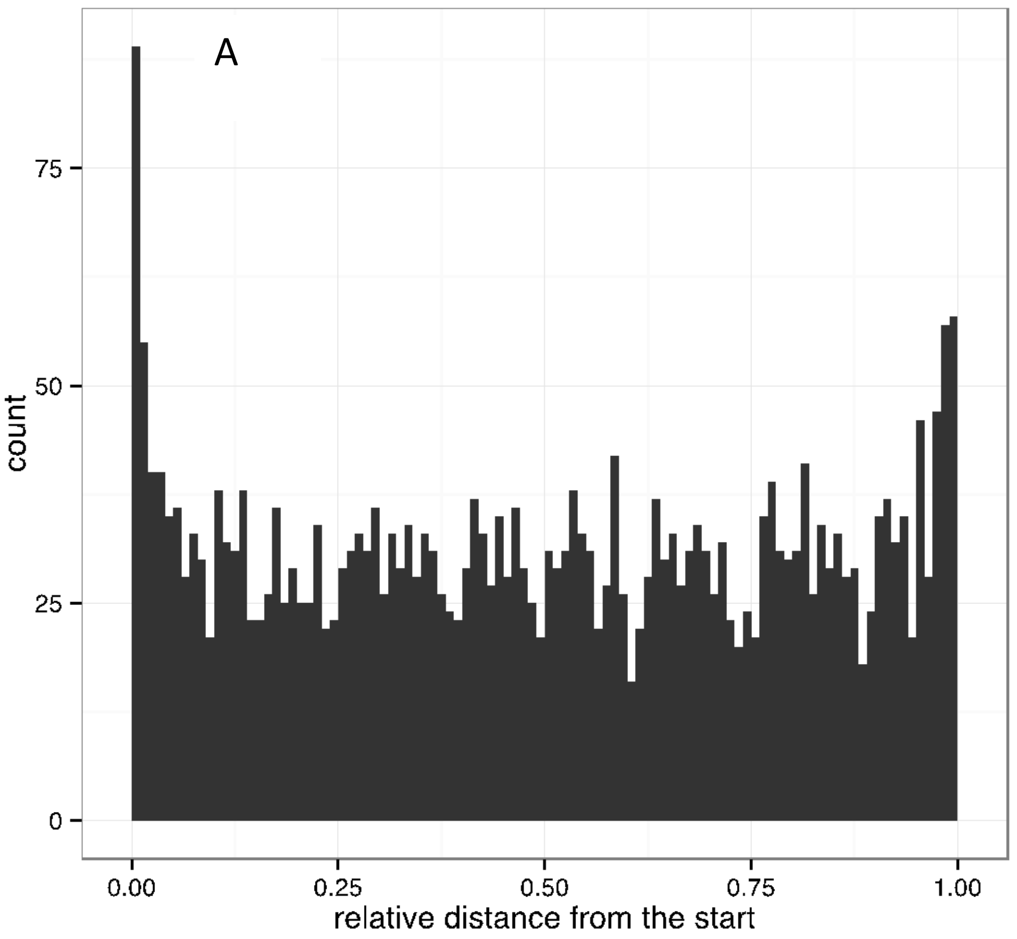

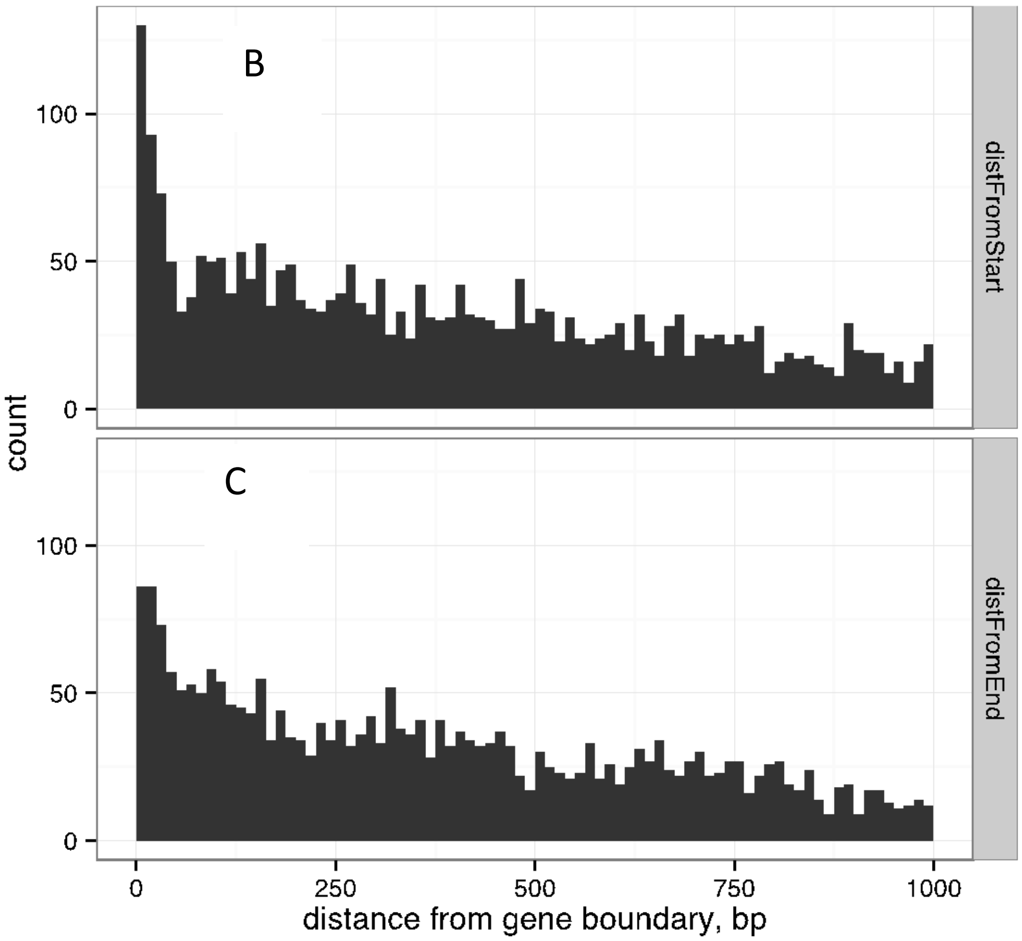

Figure IX. iTSS distribution in genes. A) Relative distance from gene start to iTSS. B) Absolute distance from gene start to iTSS. C) Absolute distance from iTSS to gene end.

Experimental procedures

Cloning procedures

Escherichia coli JM109 was used for standard cloning methods and was grown in LB broth (Yanisch-Perron et al., 1985; Sambrook et al., 1989). Plasmids were transferred from *E. coli* S17-1 to *B. japonicum* USDA 110 by biparental conjugation (Simon et al., 1982). Plasmids pJH-O1 (empty vector for overproduction of RNAs in *B. japonicum*), pJH-L1 (empty vector for transcriptional *lacZYA* fusions), pJH-F1 (vector for translational fusions to *egfp* (Andersen et al., 1998)) and their derivatives (see Additional file 11: Table S13) were constructed as previously described (Rudolf et al. 2006).

For promoter verification PCR products or annealed oligonucleotides with suitable restriction sites at the ends, which correspond to regions located upstream of mapped TSSs, were ligated into the cloning vector pME3535XhoI containing *lacZYA*. The entire transcriptional *lacZYA* fusions were then cleaved out with EcoRI and XhoI and were cloned into the broad host range vector pRK290XhoI (Morales-Alvarez et al., 1986).

For overexpression of asRNA, first the *rrn* promoter of *B. japonicum* was cloned between the EcoRI and BamHI restriction sites of pME3535XhoI. Then the resulting plasmid was used to replace the *lacZYA* genes by the *rrn* terminator of *B. japonicum* using HindIII and XhoI resulting in pJH-O1. Next, sequence corresponding to the asRNA AsR1 (complementary to blr1853 mRNA) was cloned in both orientations between BamHI and HindIII restriction sites of pJH-O1. According to RT-PCR, a putative terminator of AsR1 is located between genomic positions 2,007,288 and 2007095. Thus we cloned the region between genomic positions 2,007,288 and 2,007,669 (the latter corresponding to Bja_TSS_3939, the TSS of AsR1). Finally, the constructs containing promoter, sequence corresponding to AsR1 and terminator was cleaved out using EcoRI and XhoI and ligated into the broad host range vector pRK290XhoI.

The oligonucleotides used for cloning are listed in see Additional file 11: Table S14.

RT-PCR

Reverse transcription (RT)-PCR analyses were performed with the Tetro Reverse Transcriptase (Bioline) and Taq polymerase. 100 ng total RNA from free living cells in the exponential growth phase or 300 ng RNA from nodules were mixed with 10 pmol of the reverse primer and subjected to denaturation and annealing (5 min at 70 °C, 5 min at 50°C and 5 min at 37°C) in a 5 µl sample. Reverse transcriptase (200 u) was added together with the supplied buffer, 1 µl dNTPs (10 mM) and 40 u of the RNase inhibitor Ribolock (ThermoScientific) in a final volume of 10 µl. The sample was incubated at 45°C for 30 min for synthesis of cDNA. After inactivation of the reverse transcriptase for 10 min at 85°C, 5 µl of the cDNA-containing sample was subjected to standard PCR (30 cycles) after adding 10 pmol of each of the forward and reverse primer, the supplied buffer, 1 µl dNTPs (10 mM) and 0.25 u of Taq polymerase in a final volume of 12,5 µl. Primers used for RT-PCR are listed in see Additional file 11: Table S14.

qRT-PCR

The qRT-PCR analysis was performed with the Brilliant III Ultra-Fast SYBR Green Kit (Agilent). For strand-specific qRT-PCR, first cDNA synthesis was performed using 1 µl of the 10 mM solution of the primer complementary to the RNA of interest and 20 ng (for analysis of mRNA, asRNA or sRNA) or 0.2 ng (for the 16S rRNA reference) total RNA in a 9 µl final volume containing the master mix and the enzyme mixture of the kit. The sample was incubated for 10 min at 50°C followed by 10 min at 96 °C. Thereafter 1 µl of the 10 mM second primer corresponding to the opposite strand was added and real time PCR was performed using the BioRad Real-Time PCR Detection System CFX96 and the following program: initial denaturation for 5 min at 95°C and 40 cycles consisting of denaturation for 10 sec 95°C and annealing/elongation for 10 sec at 60°C. For normalization of mRNA, sRNA and asRNA levels, 16S rRNA was used. The relative level of an RNA under a given condition was calculated in relation to the level under other conditions and in relation to 16S rRNA (Pfaffl, 2001). Similarly, the relative level of an asRNA and mRNA was calculated including the data for 16S rRNA. Primers used for RT-PCR are listed in see Additional file 11: Table S14.

References

Alvarez-Morales A, Betancourt-Alvarez M, Kaluza K, Hennecke H. Activation of the *Bradyrhizobium japonicum nifH* and *nifDK* operons is dependent on promoter-upstream DNA sequences. Nucleic Acids Res. 1986;14:4207-27.

Andersen JB, Sternberg C, Poulsen LK, Bjorn SP, Givskov M, Molin S. New unstable variants of green fluorescent protein for studies of transient gene expression in bacteria. Appl Environ Microbiol. 1998;64:2240-6.

Aziz RK, Bartels D, Best AA, DeJongh M, Disz T, Edwards RA, Formsma K, Gerdes S, Glass EM, Kubal M, Meyer F, Olsen GJ, Olson R, Osterman AL, Overbeek RA, McNeil LK, Paarmann D, Paczian T, Parrello B, Pusch GD, Reich C, Stevens R, Vassieva O, Vonstein V, Wilke A, Zagnitko O. The RAST Server: rapid annotations using subsystems technology. BMC Genomics. 2008;9:75.

Cock PJ, Antao T, Chang JT, Chapman BA, Cox CJ, Dalke A, Friedberg I, Hamelryck T, Kauff F, Wilczynski B, and de Hoon MJ. Biopython: freely available Python tools for computational molecular biology and bioinformatics. Bioinformatics. 2009; 25:1422-3.

Ederveen, T. H. a, Overmars, L., & van Hijum, S. a F. T. Reduce manual curation by combining gene predictions from multiple annotation engines, a case study of start codon prediction. PloS One. 2013; 8: e63523.

Hemmerich C, Buechlein A, Podicheti R, Revanna KV, Dong Q. An Ergatis-based prokaryotic genome annotation web server. Bioinformatics. 2010;26:1122-4.

Hyatt D, Chen G-L, LoCascio PF, Land ML, Larimer FW, Hauser LJ. Prodigal: prokaryotic gene recognition and translation initiation site identification. BMC Bioinformatics. 2010;11:119.

Kaneko T, Nakamura Y, Sato S, Minamisawa K, Uchiumi T, Sasamoto S, Watanabe A, Idesawa K, Iriguchi M, Kawashima K, Kohara M, Matsumoto M, Shimpo S, Tsuruoka H, Wada T, Yamada M, Tabata S. Complete genomic sequence of nitrogen-fixing symbiotic bacterium *Bradyrhizobium japonicum* USDA 110. DNA Res. 2002;9:189-97.

Kearse, M., Moir, R., Wilson, A., Stones-Havas, S., Cheung, M., Sturrock, S., Buxton, S., Cooper, A., Markowitz, S., Duran, C., Thierer, T., Ashton, B., Mentjies, P., & Drummond, A. (2012). Geneious Basic: an integrated and extendable desktop software platform for the organization and analysis of sequence data. Bioinformatics. 28(12), 1647-1649.

Kingsford CL, Ayanbule K, Salzberg SL. Rapid, accurate, computational discovery of Rho-independent transcription terminators illuminates their relationship to DNA uptake. Genome Biol. 2007;8:R22

Madhugiri R, Evguenieva-Hackenberg E. RNase J is involved in the 5'-end maturation of 16S rRNA and 23S rRNA in *Sinorhizobium meliloti*. FEBS Lett. 2009;583:2339-42.

Madhugiri R, Pessi G, Voss B, Hahn J, Sharma CM, Reinhardt R, Vogel J, Hess WR, Fischer HM, Evguenieva-Hackenberg E. Small RNAs of the *Bradyrhizobium/Rhodopseudomonas* lineage and their analysis. RNA Biol. 2012;9:47-58.

Mitra A, Kesarwani AK, Pal D, Nagaraja V. WebGeSTer DB--a transcription terminator database. Nucleic Acids Res. 201139:D129-35.

Naville M, Ghuillot-Gaudeffroy A, Marchais A, Gautheret D. ARNold: a web tool for the prediction of Rho-independent transcription terminators. RNA Biol. 2011;8:11-3.

Pfaffl MW. A new mathematical model for relative quantification in real-time RT-PCR. Nucleic Acids Res. 2001;29:e45

Rudolph G, Semini G, Hauser F, Lindemann A, Friberg M, Hennecke H, Fischer HM. The Iron control element, acting in positive and negative control of iron-regulated *Bradyrhizobium japonicum* genes, is a target for the Irr protein. J Bacteriol. 2006;188:733-44.

Sambrook J, Fritsch EF, Maniatis T. Molecular cloning: A laboratory manual. 2. Cold Spring Harbor Laboratory Press, Cold Spring Harbor, NY. 1989.

Schlüter JP, Reinkensmeier J, Daschkey S, Evguenieva-Hackenberg E, Janssen S, Jänicke S, Becker JD, Giegerich R, Becker A. A genome-wide survey of sRNAs in the symbiotic nitrogen-fixing alpha-proteobacterium *Sinorhizobium meliloti*. BMC Genomics. 2010;11:245

Simon R, Priefer U, Pühler A**.** A broad host range mobilization system for *in vivo* genetic engineering: transposon mutagenesis in gram-negative bacteria. Biotechnology. 1982; 1:784-791.

Taboada B, Ciria R, Martinez-Guerrero CE, Merino E. ProOpDB: Prokaryotic Operon DataBase. Nucleic Acids Res. 2012;40:D627-31.

Tsoy OV, Pyatnitskiy MA, Kazanov MD, Gelfand MS. Evolution of transcriptional regulation in closely related bacteria. BMC Evol Biol. 2012;12:200.

Yanisch-Perron C, Vieira J, Messing J. Improved M13 phage cloning vectors and host strains: nucleotide sequences of the M13mp18 and pUC19 vectors. Gene 1985;33:103-119.
